# Rapid and Robust Generation of Homozygous Fluorescent Reporter Knock-In Cell Pools by CRISPR-Cas9

**DOI:** 10.3390/cells14151165

**Published:** 2025-07-29

**Authors:** Jicheng Yang, Fusheng Guo, Hui San Chin, Gao Bin Chen, Ziyan Zhang, Lewis Williams, Andrew J. Kueh, Pierce K. H. Chow, Marco J. Herold, Nai Yang Fu

**Affiliations:** 1 Cancer Biology and Stem Cells Division, The Walter and Eliza Hall Institute of Medical Research, Parkville, VIC 3052, Australia; yang.ji@wehi.edu.au (J.Y.); zhang.zi@wehi.edu.au (Z.Z.); 2 Department of Medical Biology, The University of Melbourne, Parkville, VIC 3052, Australia; williams.le@wehi.edu.au (L.W.); kueh@wehi.edu.au (A.J.K.); herold@wehi.edu.au (M.J.H.); 3 Cancer and Stem Cell Biology Program, Duke-NUS Medical School, Singapore 169857, Singapore; fusheng.guo@duke-nus.edu.sg (F.G.); chin.hui.san@skh.com.sg (H.S.C.); 4 Department of Medical Sciences, National Cancer Center Singapore, Singapore 168583, Singapore; chen.gao.bin@nccs.com.sg (G.B.C.); pierce.chow@duke-nus.edu.sg (P.K.H.C.); 5 Infection and Global Health Division, The Walter and Eliza Hall Institute of Medical Research, Melbourne, VIC 3052, Australia; 6 The Melbourne Genome Editing Centre (MAGEC), The Walter and Eliza Hall Institute of Medical Research, Parkville, VIC 3052, Australia; 7 Olivia Newton John Cancer Research Institute, 145 Studley Road, Heidelberg, VIC 3084, Australia; 8 School of Cancer Medicine, La Trobe University, Heidelberg, VIC 3084, Australia; 9 Surgery Academic-Clinical Program, Duke-NUS Medical School, Singapore 169857, Singapore

**Keywords:** genome editing, CRISPR-Cas9, biallelic editing, CRISPR screen, genetically engineered cells, fluorescent reporter knock-in cells, transcriptional regulation, TSPAN8

## Abstract

Conventional methods for generating knock-out or knock-in mammalian cell models using CRISPR-Cas9 genome editing often require tedious single-cell clone selection and expansion. In this study, we develop and optimise rapid and robust strategies to engineer homozygous fluorescent reporter knock-in cell pools with precise genome editing, circumventing clonal variability inherent to traditional approaches. To reduce false-positive cells associated with random integration, we optimise the design of donor DNA by removing the start codon of the fluorescent reporter and incorporating a self-cleaving T2A peptide system. Using fluorescence-assisted cell sorting (FACS), we efficiently identify and isolate the desired homozygous fluorescent knock-in clones, establishing stable cell pools that preserve parental cell line heterogeneity and faithfully reflect endogenous transcriptional regulation of the target gene. We evaluate the knock-in efficiency and rate of undesired random integration in the electroporation method with either a dual-plasmid system (sgRNA and donor DNA in two separate vectors) or a single-plasmid system (sgRNA and donor DNA combined in one vector). We further demonstrate that coupling our single-plasmid construct with an integrase-deficient lentivirus vector (IDLV) packaging system efficiently generates fluorescent knock-in reporter cell pools, offering flexibility between electroporation and lentivirus transduction methods. Notably, compared to the electroporation methods, the IDLV system significantly minimises random integration. Moreover, the resulting reporter cell lines are compatible with most of the available genome-wide sgRNA libraries, enabling unbiased CRISPR screens to identify key transcriptional regulators of a gene of interest. Overall, our methodologies provide a powerful genetic tool for rapid and robust generation of fluorescent reporter knock-in cell pools with precise genome editing by CRISPR-Cas9 for various research purposes.

## 1. Introduction

Gene-editing tools such as zinc-finger nucleases, TALENs (transcription activator-like effector nucleases), and the more recent CRISPR-Cas9 system hold significant promise for advancing gene therapy [1]. The CRISPR-Cas9 technology has enabled precise and easy modifications of the human genome, supporting profound advancements in both research and therapeutic applications. Typically, CRISPR-Cas9 components are delivered into target cells via plasmid DNA-based methods or viral infection systems. The Cas9 endonuclease induces site-specific double-strand breaks (DSBs) guided by single-guide RNAs (sgRNAs), which direct Cas9 to predefined genomic loci [2]. Mammalian cells respond to DSBs by activating two key repair pathways: non-homologous end joining (NHEJ) and homology-directed repair (HDR) [3]. NHEJ often introduces random insertions or deletions (indels) at the break site, while HDR leverages homologous DNA sequences, either from homologous chromosomes or donor templates, to precisely introduce or modify sequences at or near the DSB. Such accuracy in homology recombination is critical for precise genome engineering [4].

However, CRISPR-Cas9-mediated HDR frequently results in monoallelic knock-ins, where only one allele is precisely modified while the other remains unedited or acquires variable indels [5]. Achieving biallelic knock-in with precise editing is critical when the endogenous expression level of the gene of interest is low from a single allele, or when downstream applications such as functional analyses or screening require a homogeneous population; otherwise, mixed KI/+ and KI/KI cells can confound the interpretation and robustness of the data. Consequently, identifying and selecting cells that have undergone successful biallelic modifications is crucial for various research purposes. Single-cell cloning is a commonly used strategy to identify clones and establish cell lines with monoallelic and/or biallelic gene editing. However, this process is time-consuming and labour-intensive, and is often associated with significant drawbacks, including inherent clonal variability in the parent cells. Additionally, clonally derived reporter cells may not recapitulate the heterogeneity of the parental cells, potentially skewing experimental outcomes. To achieve HDR-directed gene editing, electroporation of plasmid DNA is an efficient transduction approach commonly used for the delivery of sgRNA plasmid and donor DNA into target cells. This delivery method remains challenging to use for many cell types and often requires large amounts of DNA, which can compromise cell viability and lead to substantial random integration [6]. In addition to this approach, electroporation of pre-assembled CRISPR-Cas9 ribonucleoprotein (RNP) complexes together with donor DNA has emerged as an effective alternative [7]. Lentiviral vectors present an ideal alternative for delivering gene-editing components due to their high transduction efficiency across diverse cell types. However, conventional lentiviral systems integrate into the host genome, posing risks of insertional mutagenesis and limiting their clinical applicability [8]. Hence, it is not feasible to deliver a donor DNA template for HDR. Additionally, the continuous expression of gene-editing components in transduced cells increases off-target effects. To address these issues, the integrase-deficient lentiviral vector (IDLV) system has recently emerged as a solution. This system enables transient delivery of sgRNA and donor DNA without genomic integration, minimising off-target and random risks while bypassing the need for electroporation [9,10]. Although a few studies support their utilities, further research is needed to fully optimise IDLV-based CRISPR-Cas9 genome editing for the generation of knock-in cells through HDR.

**Figure 1:**
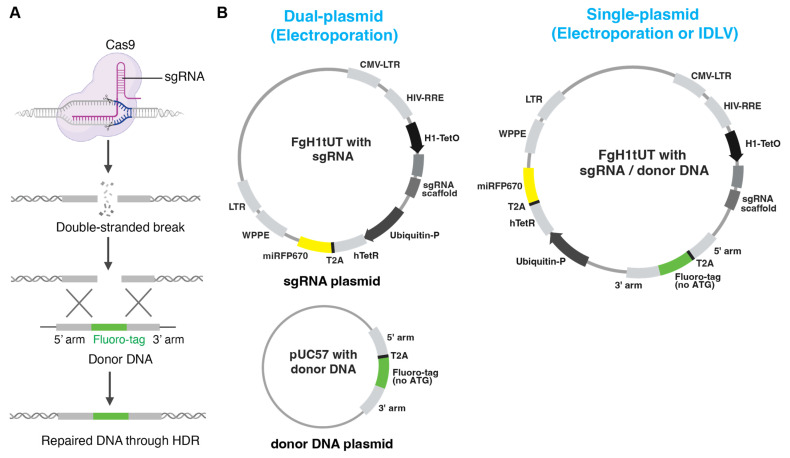
Overview of CRISPR-Cas9-based strategies for generating fluorescent reporter cell lines via in-frame knock-in. (**A**) Schematic illustration of the general strategy for generating fluorescent knock-in reporter cell lines. Cas9-expressing cells are transduced with a gene-specific sgRNA to introduce a double-stranded break at the target locus. A donor DNA template containing a fluorescent reporter is provided for homology-directed repair (HDR), enabling precise insertion of the reporter sequence into the genomic locus of interest. Created with BioRender.com. (**B**) Schematic representation of the dual-plasmid and single-plasmid systems. In the dual-plasmid system, the sgRNA and the donor DNA template are delivered by two separate vectors. The sgRNA plasmid features a doxycycline-inducible sgRNA expression cassette and a miRFP670 fluorescent reporter as a marker for transient expression of the plasmid. The donor DNA vector includes a 5′ homology arm, a fluorescent reporter in frame with a T2A peptide and lacking a start codon, and a 3′ homology arm. In the single-plasmid system, a multiple cloning site (MCS) was engineered into the sgRNA plasmid to enable subcloning of the donor DNA template. Other than expressing sgRNA induced by doxycycline, this plasmid also acts as the donor DNA for HDR within a limited time window after infection. Moreover, cells expressing the miRFP670 reporter from randomly integrated donor DNA can be eliminated by FACS sorting from a few days post-infection onwards. Created with BioRender.com.

Here, we present and compare three strategies with optimised donor DNA design for generating homozygous fluorescent knock-in cell pools: dual-plasmid and single-plasmid electroporation approaches as well as an IDLV-based delivery system (Figure 1A,B). The dual-plasmid system employs separate plasmids for donor DNA and doxycycline-inducible sgRNA expression, which are delivered via electroporation. While this approach enables efficient targeted integration of the fluorescent reporter, it is associated with higher rates of random integration. The single-plasmid system integrates both components into one vector, simplifying delivery and incorporating a fluorescent protein marker to eliminate undesired random integration. Especially, the IDLV system showed the lowest random integration while still maintaining high editing efficiency, making it particularly effective for hard-to-transfect cell types. Together, these approaches provide flexible and robust options for rapid generation of homozygous fluorescent reporter knock-in cell pools for a wide range of research purposes.

## 2. Materials and Methods

### 2.1. Cell Lines and Cell Culture

Detailed information on all commercially available and validated cell lines utilised in this study can be found in Table S1. The MEC cell line originated from the pleural effusion of a cholangiocarcinoma patient [11]. All cell lines were cultured at 37 °C tissue culture incubator with a humidified atmosphere of 5% CO_2_. All cells were cultured in Dulbecco’s Modified Eagle Medium (DMEM high glucose, GlutaMAX, ThermoFisher, Waltham, MA, USA, Cat# 10566016) supplemented with 10% (*v*/*v*) fetal bovine serum (Scientifix, Melbourne, VIC, Australia, Ref: FBSAU-2210C) and 1% (*v*/*v*) penicillin/streptomycin (ThermoFisher, Waltham, MA, USA, Cat# 15140122), except for JHH5 cells, which were maintained in William’s E media (ThermoFisher, Waltham, MA, USA, Cat# 12551032). Cells were passaged once they reached 80–90% confluency.

### 2.2. Doxycycline-Inducible sgRNA Lentiviral Vectors

To generate sgRNA plasmids for CRISPR/Cas9 genome editing, the doxycycline-inducible sgRNA vector system Fgh1tUT (Addgene, Watertown, MA, USA, Plasmid #70183) was utilised [12]. The custom construct Fgh1tUT_miRFP670 was developed in-house by replacing the eGFP gene in Fgh1tUT_GFP with the miRFP670 gene from pmiRFP670-N1 (Addgene, Watertown, MA, USA, Plasmid #79987). The single-plasmid backbone was engineered by introducing multiple cloning sites (MCS, sequence: ACCGGTTGGCGCGCCCCTGCAGGTGCTAGC) into the FgH1tUT_miRFP670 vector to facilitate the efficient subcloning of the donor DNA template into the sgRNA vector. To insert the target sgRNA sequence into a Fgh1tUT vector, two complementary oligonucleotides were synthesised with the following structure: 5′-TCCCNNNNNNNNNNNNNNNNNNNN-3′ and 3′-NNNNNNNNNNNNNNNNNNNNCAAA-5′, then annealed. All specific sgRNA sequences corresponding to the “N” regions are listed in Table S2. The vector was digested using the BsmBI restriction enzyme. The sgRNAs were either designed using Benchling (benchling.com/academic) or selected from the Sabatini/Lander CRISPR pooled library (Addgene, Watertown, MA, USA, Cat# 1000000095) as detailed in Table S2. The *TSPAN8-T2A-GFP* donor cassette was synthesised and cloned into a pUC57 vector by a commercial provider. To generate the plasmid for the single-plasmid system, the donor DNA from the pUC57 vector and sgRNA were subcloned into the sgRNA vector with MCS for cloning of donor DNA.

### 2.3. Transfection of Plasmids into Mammalian Cells by Electroporation

Transient transfections were carried out using the Invitrogen™ Neon™ Electroporation System (Thermo Fisher Scientific, Waltham, MA, USA, Cat# N10025) with the 100 µL Kit, following the manufacturer’s protocol. Briefly, the amount of plasmid DNA was used as indicated in the figures in 100 µL of Resuspension Buffer with 1 million cells. For dual-plasmid transfection, equal donor and sgRNA plasmid DNA were combined to make up the indicated total amount. Electroporation was performed with the following parameters: 870 V pulse voltage, 35 ms pulse width, and two pulses. Immediately after electroporation, cells were transferred to pre-warmed complete culture medium supplemented with 2 µg/mL doxycycline and incubated under standard culture conditions (37 °C, 5% CO_2_) for 2 days. Following this initial incubation, the medium containing transfection complexes was replaced with fresh complete medium without doxycycline. Cells were then cultured for an additional ~10 days post-transfection to minimise the potential interference of transient donor DNA expression on the analysis.

### 2.4. Production of Integrase-Competent Lentivirus (ICLV) and Infection

Lentiviral particles were generated via transient transfection of HEK 293 cells cultured in 10 cm dishes at 70% confluence using 3 μg of target plasmid DNA, 3 μg of pMDLg/pRRE, 2 μg of pMD2.G, and 2 μg of pRSV-Rev. Virus-containing supernatants were harvested 48 h post-transfection and filtered through a 0.45 μm membrane. The filtered supernatants supplemented with 2 μg/mL polybrene were added to target cells and centrifuged at 500× *g* for 30 min. Following 24 h incubation under standard culture conditions (37 °C, 5% CO_2_), the infected cells were cultured in fresh complete medium.

### 2.5. IDLV-Based Infection for Inducing Reporter Knock-In

Lentiviral particles were produced by transiently transfecting HEK 293 cells cultured in 10 cm dishes at 70% confluence with 3 μg of lentivirus plasmid DNA, 3 μg of pBK43 (psPAX2-D64E), 2 μg of pMD2.G, and 2 μg of pRSV-Rev. Virus-containing supernatants were collected 48 h post-transfection and filtered through a 0.45 μm membrane. The viral supernatants, supplemented with 2 μg/mL polybrene and 2 μg/mL doxycycline, were added to cultured cells, followed by centrifugation at 500× *g* for 30 min. Notably, doxycycline was added simultaneously with the viral supernatant to promptly induce sgRNA expression. After 48 h of incubation under standard culture conditions (37 °C, 5% CO_2_), the infected cells were re-cultured in fresh medium. Cells were then cultured for an additional ~10 days post-transfection to allow for knock-in stabilisation. To determine the multiplicity of infection (MOI), target cells were plated and infected with serial dilutions of IDLV virus expressing the miRFP670 reporter. After 48 h, cells were harvested and analysed by flow cytometry to assess miRFP670 expression. MOI = 0.5 was defined as the condition where around 50% of cells were miRFP670-positive.

### 2.6. Flow Cytometry Analysis and FACS Sorting

For FACS analysis of cell-surface TSPAN8 expression, an APC-conjugated anti-human TSPAN8 antibody was used (REAfinity™, Miltenyi Biotec, Bergisch Gladbach, Germany, Clone REA443, Cat# 130-106-811). Prior to analysis, cells were stained with 0.2 μg/mL 7-AAD (Caymanchem, Ann Arbor, MI, USA) to exclude dead cells. Flow cytometry analysis was conducted using the Fortessa cell analyser (Becton Dickinson, Franklin Lakes, NJ, USA), and cell sorting was carried out on the FACS Aria (Becton Dickinson, Franklin Lakes, NJ, USA). Data were analysed using FlowJo software (v10, Tree Star, Ashland, OR, USA).

### 2.7. Western Blot Analysis

The primary antibodies used for Western blotting are listed in Table S1. Cells were lysed in ice-cold cell lysis buffer (150 mM NaCl, 50 mM Tris-HCl, pH 7.3, 0.25 mM EDTA, pH 8.0, 1% sodium deoxycholate, 1% Triton X-100, 0.2% sodium fluoride and 0.1% sodium orthovanadate), supplemented with a protease and phosphatase inhibitor cocktail (Roche, Mannheim, Germany). The lysates were separated on 10% SDS-PAGE and transferred to PVDF membranes. After blocking with 5% non-fat milk in wash buffer (TBS, 0.01% Triton X-100, Sigma-Aldrich, Merck Pty Ltd, Castle Hill, NSW, Australia) at 37 °C for 1 h, immunoblotting was performed by incubating the membrane with primary antibodies overnight at 4 °C. The membrane was then washed three times with wash buffer and incubated with HRP-conjugated secondary antibodies for 1 h at room temperature. Protein bands were visualised using the Odyssey CLx (Image Studio 3.1 software; LI-COR Biosciences, Lincoln, NE, USA), as per the manufacturer’s instructions.

### 2.8. Polymerase Chain Reaction (PCR) Genotyping

Confirmation of the correct integration in the reporter cell lines was performed by PCR genotyping. The genomic DNA were extracted as per the manufacturer’s instructions (One-4-All Genomic DNA Miniprep Kit, Cat# BS88504, Bio Basic, Markham, ON, Canada). The PCRs were carried out on a T100 thermocycler (BioRad, Hercules, CA, USA) using the following settings: initial denaturation at 95 °C for 3 min; 28 cycles of denaturation at 95 °C for 30 s, annealing at 60 °C for 45 s, and extension at 72 °C for 1 min. A final extension step was performed at 72 °C for 5 min, followed by a hold at 4 °C until further use. The specific PCR primers used are listed in Table S3.

### 2.9. Genome-Wide CRISPR/Cas9 Knockout Screening

The Human Two Plasmid Activity-Optimized CRISPR Knockout sgRNA Library, developed by David Sabatini, Eric Lander and colleagues, was obtained from Addgene (Cat# 1000000095, Watertown, MA, USA) [13]. Lentiviral particles were produced by packaging the library into HEK 293 cells. Pilot experiments were performed to determine the virus input required to achieve MOI = 0.3–0.5, corresponding to around 70% and 50% cell death upon puromycin selection, respectively, as previously described [14]. For screening, cells were seeded in T150 flasks and cultured prior to infection. Approximately 300 million cells were exposed to lentivirus in a medium supplemented with 2 μg/mL polybrene at an MOI of 0.3–0.5 for two days. Infected cells were then selected with 10 µg/mL puromycin in fresh media for two days and subsequently cultured in puromycin-free fresh media for several days before FACS sorting. A total of 10–20% of the total infected cells were reserved as the pool control sample, and the remaining 80–90% of cells were used for FACS sorting based on GFP expression. The genomic DNA was extracted from both the pooled control and sorted cells according to the manufacturer’s instructions (Bio Basic, One-4-All Genomic DNA Miniprep Kit, Markham, ON, Canada). NGS libraries were generated via PCR using specific barcode primers. The sgRNA barcode PCR primers we as follows: Forward: CAAGCAGAAGACGGCATACGAGATCnnnnnnTTTCTTGGGTAGTTTGCAGTTTT; Reverse: AATGATACGGCGACCACCGAGATCTACACnnnnnnnnCACCGACTCGGTGCCACTTTT. “n” represents sample-specific barcode sequences. Multiplexed NGS libraries were sequenced on the HiSeq4000 platform (Illumina, San Diego, CA, USA) to identify the sgRNAs in each sample. Data normalisation and sgRNA modelling and ranking were performed by using the MAGeCK algorithm as previously described [15].

### 2.10. Generation of CRISPR-Cas9 Knockout Cells

To generate sgRNA-induced gene knockout cells, a two-plasmid lentiviral sgRNA inducible expression system was employed, which consists of (1) FuCas9Cherry vector (Addgene#70182, Watertown, MA, USA), which constitutively expresses Cas9 and the mCherry fluorescence marker, and (2) FgH1tUT vector, which drives doxycycline-inducible sgRNA expression along with constitutive expression of either eGFP or TagBFP, as previously described. Cells were first transduced with the pFU_Cas9_mCherry plasmid and sorted using the FACS Aria (Becton Dickinson, Franklin Lakes, NJ, USA) to establish the stable Cas9_mCherry expressing populations. The Cas9 stable cells were then infected with lentivirus carrying doxycycline-inducible sgRNAs (Fgh1tUT vectors) targeting coding exons of the gene of interest. To activate the sgRNA expression via the Tet-O promoter, cells were treated with 1ug/mL doxycycline at 37 °C in a humidified incubator with 5% CO_2_ for two days. Following that, cells were washed with PBS and cultured in a fresh and doxycycline-free complete medium for a minimum of five days. KO populations were enriched by FACS, selecting the cells double positive for GFP and BFP.

### 2.11. Reverse Transcription and qPCR Analysis

Total RNA was extracted following the manufacturer’s protocol (Qiagen, RNeasy Micro Kit, Cat# 74004, Hilden, Germany). mRNA was reverse-transcribed into cDNA with GoScriptTM Reverse Transcription Kit (Promega, Cat# A5000, Madison, WI, USA). The resulting cDNA was used for either gene cloning via PCR amplification or for qPCR. qPCR was performed using SYBR Green qPCR Master Mix (Thermo Fisher Scientific, Waltham, MA, USA) with specific forward and reverse primers. Reactions were run on a Bio-Rad System (Bio-Rad, Hercules, CA, USA). Relative mRNA expression levels were calculated by the standard delta-delta Ct (2^−∆∆Ct^) method, with *β-actin* serving as the housekeeping control.

The *TSPAN8* primers used for qPCR were as follows: Forward: GCAGAGACCATGCCAAAGCTATAATG and Reverse: CGATCTGGCAATACAGGACCATAG. *β-actin* primers used for qPCR; Forward: TCCCTGGAGAAGAGCTACG and Reverse: GTAGTTTCGTGGATGCCACA.

### 2.12. Long-Range PCR for TSPAN8

Genomic DNA was extracted using the Qiagen DNeasy Kit (Qiagen, Hilden, Germany) according to the manufacturer’s protocol. Long-range PCR amplification of the TSPAN8 locus was performed using Takara PrimeSTAR GXL DNA Polymerase (Cat# R051A, Takara Bio, Kusatsu, Shiga, Japan). PCR cycling conditions were as follows: 95 °C for 2 min, followed by 30 cycles of 95 °C for 30 s and 68 °C for 8 min, with a final extension at 68 °C for 5 min. Two primer sets for the TSPAN8 genomic region spanning the sgRNA targeting site were used: Set 1—forward primer F1: 5′-GTCTATAACCTGCCCTCCCTCTTTTTAAGG-3′ and reverse primer R1: 5′-GCAAAAGAAACTACCATCAGAGTGAACAGG-3′; Set 2—forward primer F5: 5′-TGAATGACCTCTCTACCGGGAAAAAGATAG-3′ and reverse primer R5: 5′-CATAACGAAATGAAGGCAGAAATGAAGATG-3′. PCR products were analysed by agarose gel electrophoresis.

### 2.13. Droplet Digital PCR (ddPCR)

Digital PCR was performed using the Qiagen QIAcuity Four system with genomic DNA extracted using the Qiagen DNA extraction kit (Qiagen, Hilden, Germany) and quantified by the high-sensitivity dsDNA quantification kit for Qubit (Invitrogen, Waltham, MA, USA). The same amount of genomic DNA for each sample was digested with the 4-cutter restriction enzyme AluI and carried forward into the ddPCR reaction. For detection of eGFP integration, the following primers and probe were used: forward primer GACAACCACTACCTGAGCAC, reverse primer CAGGACCATGTGATCGCG, and probe CCTGAGCAAAGACCCCAACGAGAA labelled with 6-FAM (5′), ZEN (internal quencher), and Iowa Black FQ (3′). For quantification of the upstream 5′ homology arm of the human TSPAN8 locus, the assay included forward primer GATGATCAGCACTTTCCTTGC, reverse primer ACTGCTTTATTTCTAGGACCTCC, and probe TGATGGCTCTCAGTGTGTAGCACTTTT labelled with Texas Red-X (5′) and Iowa Black RQ-Sp (3′). Thermal cycling was conducted using the standard probe-based duplex protocol provided by Qiagen (Hilden, Germany).

### 2.14. Quantification and Statistical Analysis

Unless otherwise specified, data are presented as mean ± standard error of the mean (SEM), and the significance was assessed by unpaired *t*-test using GraphPad Prism (v10, GraphPad Software, San Diego, CA, USA), with *p* < 0.05 considered significant.

## 3. Results

### 3.1. FACS Is an Efficient Way to Enrich Biallelically Engineered Knock-In Reporter Cells

To integrate a fluorescent reporter into the genome by CRISPR-Cas9 genome editing, we first tested a dual-plasmid system, as commonly employed in previous studies [16,17,18,19,20]. In this approach, the sgRNA targeting the desired genomic locus and the donor DNA for HDR were cloned into two separate vectors. We used the FgH1tUT vectors, which enable the sgRNA expression under the control of doxycycline and harbour the miRFP670 fluorescent reporter driven by the Ubiquitin promoter [21] (Figure 1B). The donor DNA, cloned into the pUC57 vector, contains the *T2A-GFP* [22] cassette flanked by 5′ and 3′ homology arms (~800 bp) at both sides. It is important to note that the start codon of the GFP reporter was removed to minimise GFP expression from out-of-frame and random integrations.

**Figure 2:**
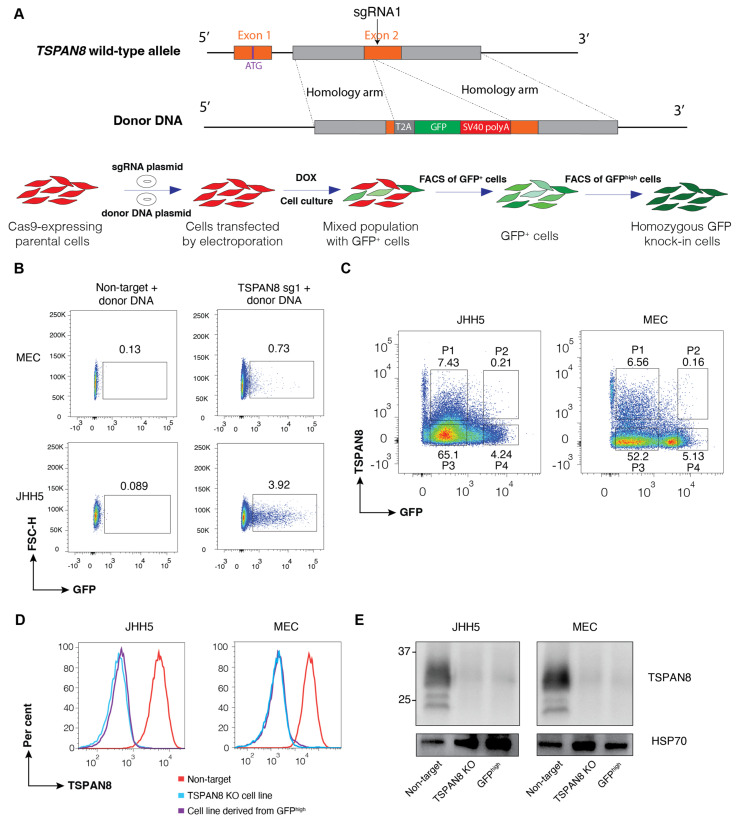
Generation of biallelic knockout cells with fluorescent knock-in reporter as a surrogate of gene disruption. (**A**) Workflow for generating TSPAN8 knock-out cell lines with a GFP knock-in reporter. The donor DNA template consists of an 800 bp 5′ homology arm, T2A-GFP sequence, SV40 polyA signal, and an 800 bp 3′ homology arm flanking exon 2 of the *TSPAN8* gene. The TSPAN8 sgRNA target site was abolished in this donor template to prevent further DNA breaks after integration. Cas9-expressing cells were transfected with dual plasmids (sgRNA and donor template) by electroporation. Immediately following transfection, cells were treated with doxycycline (Dox) to induce sgRNA expression for 48 h. After 7–10 days of culture, cells were enriched based on GFP expression levels to isolate targeted populations. (**B**) Representative FACS plot showing the initial sorting of cells. Only cells transfected with TSPAN8 sgRNA exhibit a GFP^+^ population, confirming successful targeting and reporter integration. The GFP^+^ population was gated and sorted for subsequent analyses and enrichment. Both MEC and JHH5 cells were used in this experiment, with 20 µg of total plasmids (1:1 ratio of sgRNA and donor template plasmids) transfected per million cells. (**C**) FACS plot showing TSPAN8 protein expression in relation to GFP expression in the initially sorted cell population (GFP^+^ cells gated in (**B**)). Notably, the majority of GFP^high^ cells exhibited an absence of TSPAN8 expression, confirming the efficacy of the reporter enrichment strategy. GFP^high^ cells were sorted to further enrich the homozygous reporter cell population and establish the final cell line. (**D**) FACS analysis demonstrates that most cells in the final homozygous cell line derived from the sorted GFP^high^ population were TSPAN8-negative. (**E**) Western blotting validates the absence of TSPAN8 protein in the homozygous reporter cell line. An established TSPAN8 knockout (KO) and non-targeting (NT) cells generated in our previous study were used as negative and positive controls, respectively, for TSPAN8 expression.

We selected *TSPAN8*, a gene encoding a cell-surface protein [23,24], as a representative example for our studies. We designed a sgRNA targeting Exon 2 of *TSPAN8* and constructed a corresponding donor DNA template for HDR to delete the endogenous TSPAN8 protein, while introducing a T2A-GFP reporter and SV40 polyadenylation (polyA) signal to terminate its transcription (Figure 2A). We tested this strategy in the liver cancer cell lines MEC and JHH5, which express endogenous TSPAN8 and have been engineered to stably express Cas9 [23]. The sgRNA and donor DNA plasmids were simultaneously transduced into the cells by electroporation. Subsequently, the expression of sgRNA was induced by doxycycline treatment to create TSPAN8 knockout cells with the expression of fluorescent reporter as a surrogate for successful HDR. In parallel, a non-target sgRNA plasmid was co-transduced with the donor DNA plasmid into cells as a negative control. Compared to the non-target sgRNA control, a significant proportion of cells transduced with the *TSPAN8* sgRNA expressed GFP, suggesting successful HDR (Figure 2B). GFP-positive cells were enriched by FACS and cultured for one week. Notably, most of the GFP^low^ cells displayed reduced or absent expression of cell-surface TSPAN8 protein (Figure 2C), suggesting monoallelic reporter integration with NHEJ-mediated indels in the second allele of these cells. We then conducted two rounds of FACS, separated by 6 days of cell culture, to enrich the top ~5% GFP^high^ cells and establish TSPAN8 KO reporter JHH5 and MEC cell lines, which exhibited complete loss of TSPAN8 expression, confirmed by FACS (Figure 2D) and Western blot (Figure 2E) analyses. Collectively, these data suggest that the higher reporter intensity is well correlated with biallelic editing, which enables rapid isolation of precisely engineered cell pools.

### 3.2. Generation of Homozygous Fluorescent Knock-In Reporter Cell Pools Using a Dual-Plasmid System

**Figure 3:**
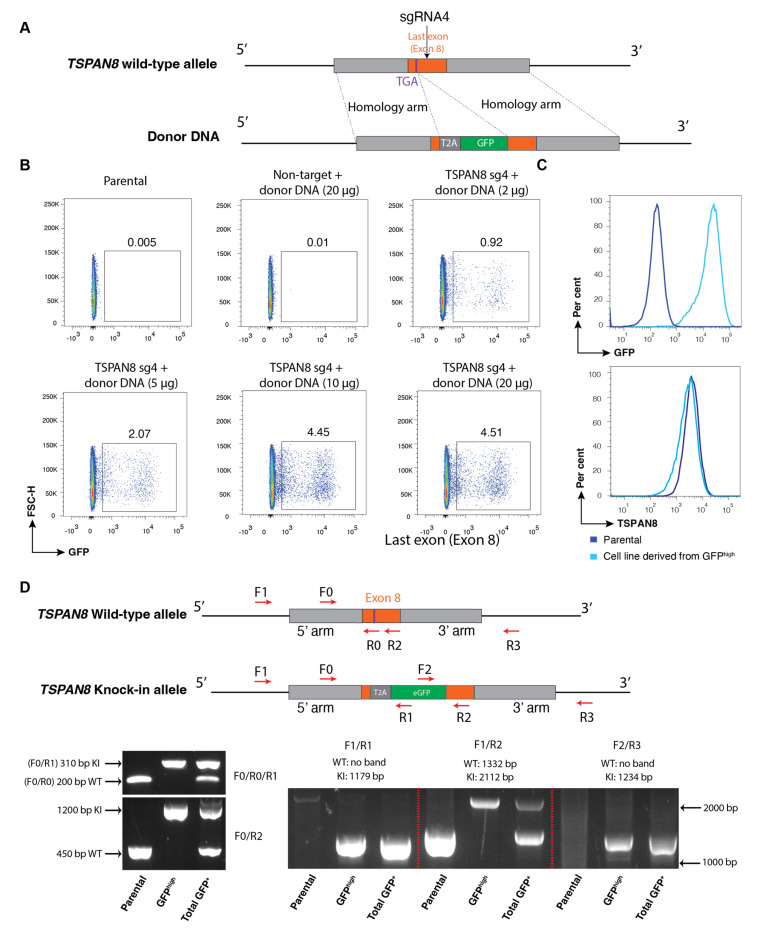
Establishment of biallelic knock-in reporter cells retaining endogenous protein expression. (**A**) Schematic illustration of the targeting strategy for the *TSPAN8* gene locus by CRISPR-Cas9. In this strategy, the stop codon of the human *TSPAN8* gene is replaced with a *T2A-eGFP* sequence. The resulting mRNA from the modified allele encodes two separate proteins: TSPAN8 and GFP. (**B**) Representative FACS analysis of electroporated cells. Different dosages of plasmids were tested on MEC cells. Notably, in the absence of TSPAN8 sgRNA (i.e., non-target sgRNA), no GFP-positive cells were observed. (**C**) Representative FACS plots of the reporter cell line generated after multiple rounds of sorting of the GFP^high^ population. The established reporter cell line exhibits high GFP and intact TSPAN8 expression. (**D**) PCR genotyping analysis of the established reporter cell line. The total GFP^+^ cells exhibited two bands corresponding to knock-in (KI) and wild-type (WT) alleles, respectively. The WT allele is absent in the reporter cell line established from the GFP^high^ population.

Next, we adapted the dual-plasmid electroporation approach to develop fluorescent knock-in reporter cell pools while preserving endogenous gene expression. We designed an sgRNA targeting the sequence near the stop codon of the *TSPAN8* gene. We engineered the donor DNA plasmid with the *T2A-eGFP* cassette flanked by 5′ and 3′ homology arms, replacing the stop codon. Of note, the *SV40 polyA* sequence was omitted to preserve the endogenous 3′ untranslated region (3′UTR) of *TSPAN8* (Figure 3A). Different plasmid dosages (e.g., 2, 5, 10, 20, and 30 µg per million cells) were electroporated into the MEC cells. Among these, the 10 and 20 µg plasmid yielded the maximum efficiency for GFP^+^ cells (~4%) (Figure 3B), with higher doses causing significant cell death. These findings align with previous studies showing that overly high plasmid concentrations can lead to cellular toxicity, reduced transfection efficiency, or activation of cellular stress responses to impair HDR efficiency [20,25]. Following electroporation, we performed the first round of FACS to isolate all the GFP^+^ cells. After culture and expansion, we sorted again the top ~10% GFP-expressing cells. We also conducted single-cell sorting to establish clonal lines for evaluation. Genotypic analysis of these clones revealed that ~75% of them most likely harboured homozygous knock-in (KI/KI) alleles (Figure S2B). Flow cytometry analysis showed KI/KI clones expressed higher levels of GFP compared to heterozygous (KI/+) (Figure S2B). To establish a cell pool with biallelic integration, the top ~5% of the GFP^high^ cells derived from the second sorting were enriched again and expanded to establish the final homozygous GFP knock-in cell pool line. FACS analysis confirmed that the endogenous expression of TSPAN8 protein remains intact in these cells (Figure 3C).

PCR amplification using primers flanking the target region, followed by Sanger sequencing, is commonly employed to detect the correct integration of the tag into the target locus [16,26,27]. To confirm biallelic integration of GFP reporter at the *TSPAN8* locus, we designed specific primers and performed PCR genotyping using genomic DNA extracted from GFP^+^ (total GFP-positive) and GFP^high^ (top ~5% GFP-expressing) cell populations, in comparison to the parental cells. The GFP^+^ cells exhibited bands corresponding to both knock-in (KI) and wild-type alleles. In contrast, only the KI band was detected in the final homozygous GFP knock-in cell pool line derived from the GFP^high^ cells, confirming the presence of biallelic GFP insertion (Figure 3D). Sanger sequencing of the PCR-amplified target region further confirmed the precise insertion of the T2A-eGFP cassette into the intended locus of the cell line (Figure S1E). We also used a sgRNA targeting the TSPAN8 exon to confirm that knockout of TSPAN8 significantly reduced GFP expression, indicating that GFP accurately reflects TSPAN8 expression (Figure S2C).

### 3.3. An Improved Single-Plasmid System for Generation of Fluorescent Knock-In Reporter Cell Pools

While our dual-plasmid system allows identification and elimination of randomly integrated sgRNA plasmids through miRFP670 reporter expression, potential undesired random integration of the donor DNA plasmid remains a concern. Although the randomly integrated donor DNA is unlikely to express a functional GFP reporter due to the lack of promoter and the start codon, we sought to directly assess donor plasmid integration events. We designed two pairs of primers targeting the upstream and downstream sequences of the donor DNA in the pUC57 vector, respectively. PCR genotyping analysis revealed that random integration occurred in both GFP^+^ and GFP^high^ reporter cell populations (Figure S1A).

**Figure 4:**
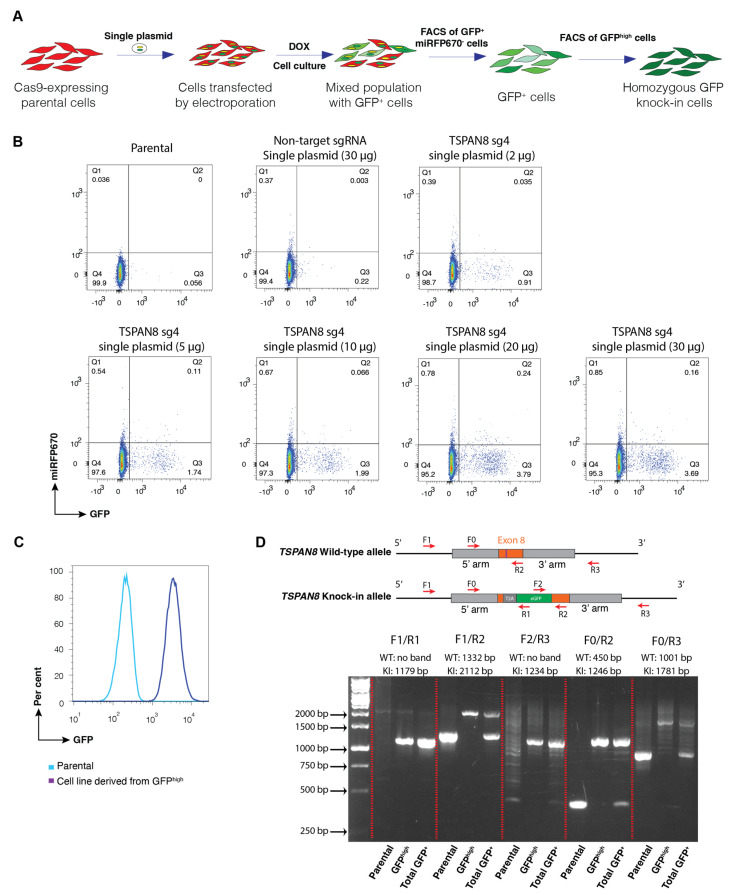
Generation of knock-in reporter cells using a single-plasmid system integrating both sgRNA and donor DNA. (**A**) Workflow for generating GFP knock-in cell lines using a single plasmid. Cas9-expressing MEC cells were transfected with the single plasmid by electroporation and cultured for 7–10 days. GFP^+^/miRFP670^−^ populations were enriched by FACS. (**B**) Representative FACS plots for cells electroporated with different amounts of single plasmid and analysed 10 days post-transduction. Transduced cells with the single plasmid lacking TSPAN8 sgRNA (i.e., non-target sgRNA) were also tested as a negative control. (**C**) Representative FACS plots of the knock-in reporter cell line generated after multiple rounds of GFP^high^ sorting. (**D**) PCR genotyping of the established reporter cell line. The total GFP^+^ cells exhibited bands corresponding to both knock-in (KI) and wild-type (WT) alleles. The WT allele is absent in the reporter cell line derived from the GFP^high^ population.

To streamline the genome-editing process and reduce random integration events, we developed an optimised single-plasmid system by directly incorporating the donor DNA into the FgH1tUT sgRNA plasmid (Figure 1B). In this system, miRFP670 serves as a marker for random plasmid integration. Following plasmid transfection into cells by electroporation, the GFP^+^/miRFP670^−^ population was sorted by FACS to enrich cells with integration of GFP reporter into the *TSPAN8* locus (Figure 4A). Similarly, 2, 5, 10, 20, and 30 µg plasmid DNA per million cells was transfected into MEC cells by electroporation. The dosage with 20 µg plasmid yielded the maximum efficiency for GFP-positive cells (~3.8%) (Figure 4B), which is slightly less than that by the dual-plasmid system (4.5%, Figure 3B). We enriched the GFP^high^ cell population through two rounds of FACS to establish the homozygous knock-in cell pool line (Figure 4C) using a similar strategy as described above for the dual-plasmid system.

To validate biallelic GFP integration at the *TSPAN8* locus, we also used different sets of specific primers and conducted PCR genotyping. Consistent with results from the dual-plasmid system, the GFP^+^ population exhibited bands corresponding to both the KI and wild-type alleles. In contrast, only the KI band was detected in the final homozygous GFP knock-in cell pool line derived from the GFP^high^ population, suggesting that the cell line most likely harbours correct biallelic insertion of the GFP reporter (Figure 4D).

### 3.4. Generation of Fluorescent Knock-In Reporter Cells by the Integrase-Deficient Lentivirus Vector System

The integrase-deficient lentivirus vectors (IDLVs) represent a significant advancement in gene-delivery technology, as they provide a safe and effective means for transient expression without genome integration (Figure S1B). In the third-generation integration-competent lentivirus vector (ICLV) system [23], lentivirus particles are produced using three packaging plasmids: pM2D.G, pRSV-Rev and pMDLg/pRRE. To generate IDLV lentivirus, the packaging plasmid pMDLg/pRRE was replaced with the pBK43 (D64E) plasmid, which disables the integration function of lentiviral vectors while maintaining their ability to efficiently deliver genetic materials into infected cells [28]. To confirm the transient expression characteristic of the IDLV system, we generated IDLV and ICLV lentiviruses expressing a BFP reporter in parallel. At 48 h post-infection, FACS analysis confirmed BFP expression in infected cells, with ICLV-infected cells displaying brighter BFP fluorescence. By day 7, ICLV-infected cells maintained BFP expression, consistent with stable genomic integration. In contrast, BFP expression was nearly undetectable in IDLV-infected cells (Figure S1C).

**Figure 5:**
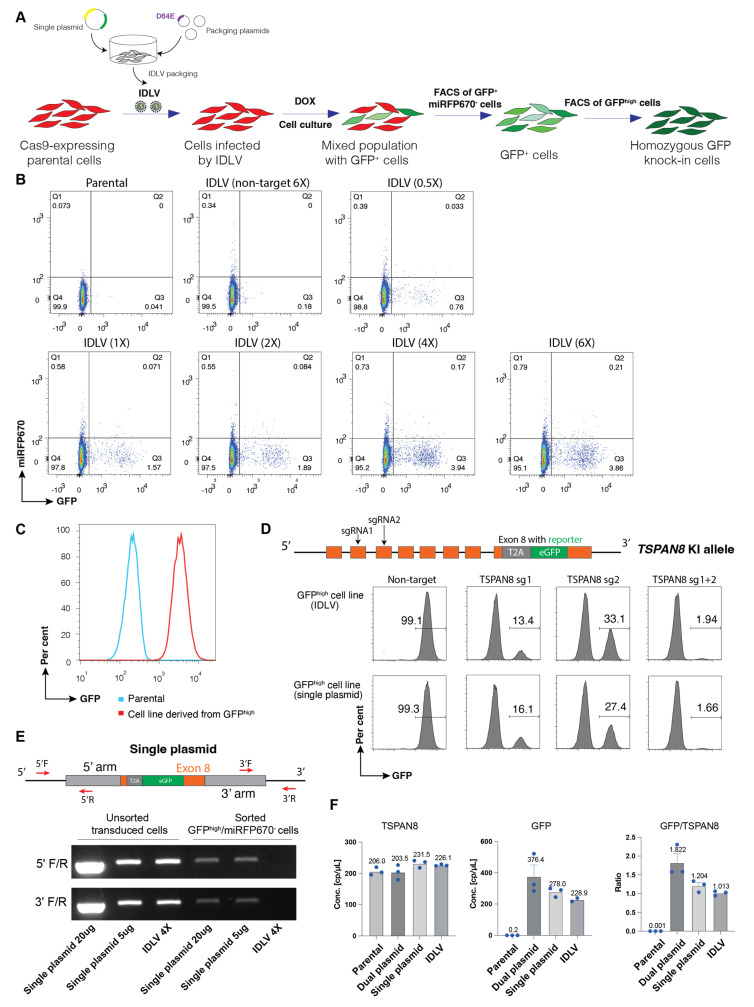
Generation of knock-in reporter cells by the IDLV system. (**A**) Workflow for generating GFP knock-in cell lines using the IDLV system. Cas9-expressing MEC cells were infected with lentivirus packaged by the IDLV system. Following infection, cells were treated immediately with doxycycline to induce sgRNA expression. GFP^+^/miRFP670^−^ populations were enriched by FACS. (**B**) Representative FACS plots of cells at 10 days post-infection with the indicated amount of lentivirus generated by the IDLV system. Lentivirus lacking sgRNA was used as a negative control. (**C**) Representative FACS plots showing a uniform GFP expression in the established knock-in reporter cells, established by multiple rounds of sorting of the GFP^high^ population. (**D**) Validation of correct integration of GFP reporter into the *TSPAN8* locus. Two distinct sgRNAs targeting early exons of *TSPAN8* were used for validation. (**E**) PCR analysis of random integration. PCR analysis was conducted using indicated primers targeting the sequences spanning the plasmid backbone and homology arms. Unsorted cells after transduction or the established reporter cell lines by single-plasmid electroporation or IDLV were analysed. (**F**) Droplet digital PCR (ddPCR) analysis of GFP and TSPAN8 was performed to determine their genomic copy numbers. Primers targeting the GFP insert and the endogenous TSPAN8 locus were used to assess the relative abundance of each sequence. Each sample was analysed in triplicate.

Building on our single-plasmid design, we adapted this system for IDLV delivery to generate homozygous GFP knock-in cell pools by HDR (Figure 5A). We tested different doses of IDLV with 1X dosage corresponding to the viral input required to achieve a multiplicity of infection (MOI) of 1. Notably, increasing the IDLV dosage to 4X resulted in an integration efficiency comparable to the maximum achieved through the single-plasmid transfection system, with no further improvement at 6X, suggesting system saturation (Figure 5B). We first enriched the total GFP^+^ cells and subsequently isolated the top ~5% GFP^high^ population by FACS sorting to establish a reporter cell pool line (Figure 5C).

To further validate the genomic integration accuracy and functional coupling of the GFP reporter to the endogenous *TSPAN8* gene, we designed two distinct sgRNAs (sgRNA1 and sgRNA2) targeting the early exons 2 and 3 of *TSPAN8*, respectively, to generate KO cells. Infection with either sgRNA1 or sgRNA2 lentivirus alone profoundly abolished the GFP expression in ~70–80% of cells, while co-infection with both sgRNAs abolished GFP expression in nearly the entire cell population. These results suggest that the GFP reporter was accurately integrated into the *TSPAN8* locus in all cells (Figure 5D). PCR genotyping verified that the GFP^high^ cell pool line generated by the IDLV system harbours biallelic integration of the reporter into the *TSPAN8* locus (Figure S1D). Of note, our dual-selection strategy (GFP^high^/miRFP670^−^) effectively minimised cells with random integrations, regardless of delivery method (electroporation or IDLV infection). While random integration of the plasmid was detectable in the unsorted total cell population, it was significantly eliminated in the FACS-sorted GFP^high^/miRFP670^−^ cell pools. Importantly, even at a 4X IDLV dosage, random integration was undetectable in the homozygous GFP reporter KI cell pools (Figure 5E). These results underscore the advantage of the IDLV delivery system over electroporation-based methods in minimising random plasmid integration. To further confirm that the absence of the wild-type allele is not due to deletion of the targeted TSPAN8 locus, long-range PCR was performed (Figure S3A). In parallel, droplet digital PCR (ddPCR) targeting sequences upstream of the TSPAN8 5′ homology arm and within the GFP insert (Figure 5F and Figure S3B) revealed that the copy number of TSPAN8 remained constant between parental and reporter knock-in cells. Interestingly, in cells generated using the dual and single plasmid electroporation approaches, the GFP:TSPAN8 copy number ratios were elevated (1.8 and 1.2, respectively), indicating varying degrees of random GFP integration. In contrast, cells generated using the IDLV method exhibited a near 1:1 GFP:TSPAN8 ratio, suggesting negligible random integration. These data are consistent with other analysis approaches, further supporting the specificity and precision of the IDLV-based approach.

### 3.5. Genome-Wide CRISPR-Cas9 Screening Identifies Transcriptional Regulators of TSPAN8

**Figure 6:**
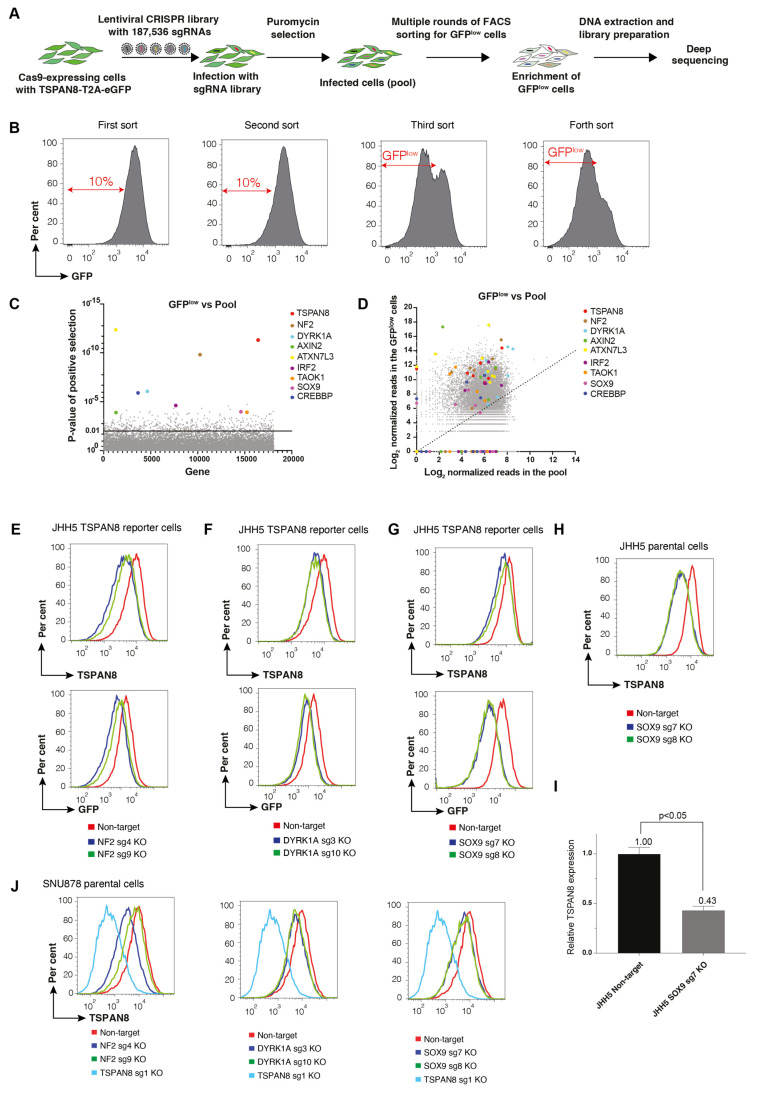
Genome-wide CRISPR-Cas9 screen for transcriptional regulators of TSPAN8. (**A**) Schematic illustrating the workflow of the genome-scale CRISPR/Cas9 loss-of-function screen to enrich the GFP^low^ cells. JHH5 reporter cells stably expressing Cas9 were transduced with a lentiviral sgRNA library. The GFP^low^ cell population was enriched through repeated rounds of sorting and culture. The enriched sgRNAs were identified via next-generation sequencing (NGS). (**B**) FACS plots showing sequential enrichment of the cells with reduced GFP expression. The GFP^low^ cells were sorted over multiple rounds, with one week of expansion between rounds. (**C**) Candidate genes were identified by comparing the sgRNA read counts between the initial pool and the final GFP^low^ population. (**D**) Scatterplot displaying the sgRNA enrichment for selected candidate genes. (**E**–**G**) Representative FACS plots showing the reduction in both cell-surface TSPAN8 (**E**) and GFP reporter expression (**G**) following knockout of NF2, DYRK1A, and SOX9. (**H**,**I**) FACS and qPCR analysis showing that knockout of SOX9 in JHH5 parental cells significantly downregulates TSPAN8 expression. (**J**) Representative FACS plots showing knockout of NF2, DYRK1A, and SOX9 reduced cell-surface TSPAN8 expression in the human HCC cell line SNU878.

The *T2A-GFP* reporter cassette in our knock-in strategy preserves native transcriptional control while providing a quantitative fluorescence readout of target gene expression. Using the JHH5 TSPAN8-T2A-eGFP knock-in reporter line, we conducted genome-wide CRISPR-Cas9 screening to systematically identify transcriptional regulators of *TSPAN8*. We performed the screen using a genome-wide lentiviral sgRNA library (~200,000 sgRNAs, 10 per gene) on approximately 300 million GFP reporter cells to ensure sufficient coverage for robust identification of transcriptional regulators of *TSPAN8* (Figure 6A). We aimed to enrich cells exhibiting reduced GFP expression, each time isolating the bottom 10% GFP^low^ population by FACS sorting, followed by 5–7 days of culture and expansion, until a distinct GFP^low^ population emerged (Figure 6B). In our screen, most sgRNAs targeting *TSPAN8* in the library were significantly enriched in the GFP^low^ population, providing strong evidence of the screen’s effectiveness (Figure 6C,D). To further confirm the screen’s results, we selected several top candidate genes (Table S4) for validation by individual sgRNAs targeting those genes. Notably, sgRNAs targeting NF2, DYRK1A, and SOX9 each led to reduced cell-surface TSPAN8 expression and GFP reporter expression (Figure 6E–G). We further validated that knockout of SOX9 significantly downregulated TSPAN8 expression in JHH5 parental cells by both FACS and qPCR analyses (Figure 6H,I). Moreover, knockout of NF2, DYRK1A, and SOX9 in another TSPAN8-expressing human liver cancer cell line, SNU878, similarly resulted in a downregulation of TSPAN8 (Figure 6J). Notably, SOX9 has been previously identified as a transcription factor regulating TSPAN8 [29], further supporting the robustness of our approach.

## 4. Discussion

CRISPR-Cas9 genome editing has revolutionised the ability to engineer specific loci in cultured cells. However, the laborious process of isolating clonal populations with desired modifications remains a major bottleneck. This problem becomes even more challenging when attempting to generate homozygous knock-in reporter cell lines via HDR, where low biallelic editing efficiencies typically yield predominantly heterozygous clones. In some cases, two rounds of iterative CRISPR tagging may be necessary to achieve homozygosity. In this study, we present optimised strategies for rapid and robust generation of homozygous fluorescent knock-in reporter cell pools using CRISPR-Cas9, which are particularly valuable for studying gene regulation and function, especially in cancer cell models where inherited heterogeneity poses a key challenge.

Our approach is based on the principle that cells with biallelic knock-ins display roughly twice the fluorescence intensity of those with monoallelic knock-ins, enabling reliable discrimination between the two populations. the fluorescent reporter intensity in cells with biallelic knock-in is approximately twice that of cells with monoallelic knock-in. Hence, a pool of the rare homozygous fluorescent knock-in clones can be rapidly isolated by FACS from a large population of the target cells to establish a homozygous knock-in reporter cell line. Eliminating the background expression of the fluorescent reporter from random integration is crucial for the success of the protocol. To ensure the expression of GFP is only from the locus with correct integration of the donor DNA through HDR, we used the T2A system and removed the start codon of the fluorescent reporter in the donor DNA design. Moreover, we utilised a doxycycline-inducible system to transiently switch on the expression of sgRNA. The data clearly demonstrate tight doxycycline control for both plasmid electroporation and IDLV-mediated delivery: successful gene editing, indicated by the expression of reporter, occurred only in the presence of doxycycline (Figure S2A). We validated the feasibility and efficiency of our strategy by generating TSPAN8 knockout cells, using knock-in GFP reporter expression as a surrogate, through co-transfection of two separate plasmids encoding the sgRNA and donor DNA via electroporation. Our results clearly show that cell clones with precise biallelic integration of GFP can be identified and isolated by FACS based on higher expression of the knock-in reporter. It is important to note that the large proportion of GFP^low^ cells also exhibited a complete absence of the TSPAN8 protein. However, unlike the GFP^high^ knockout cells, which underwent precise genomic editing, these GFP^low^ cells likely harbour a large variety of out-of-frame indels in the second allele, which is not ideal for certain downstream utilities of knock-out cells.

We next applied a similar approach to generate knock-in reporter cell pools by replacing the stop codon of a target gene with the T2A-GFP cassette, thereby preserving the endogenous transcriptional regulation of the target gene as much as possible. For these cell lines, we confirmed successful biallelic knock-in using PCR genotyping with multiple specific primer sets. To further streamline our method, we developed a single-plasmid system in which both the sgRNA expression cassette and donor DNA template for HDR are incorporated into a single vector. Our data demonstrated that sgRNA was successfully induced to mediate the cleavage of the desired gene locus, while the plasmid served as the donor DNA for the efficient integration of the reporter through HDR. The single-plasmid electroporation system lowers the risk of random integration by incorporating the miRFP670 reporter, allowing exclusion of undesired cells; however, it exhibits slightly lower HDR efficiency compared to the dual-plasmid system.

It is well known that certain cell types are resistant to transfection or electroporation, and often exhibit high levels of cell death following these procedures. In contrast, the lentivirus infection as a tool for the delivery of genetic materials shows high efficiency and low cytotoxicity across a wide range of cancer and primary cells. However, lentivirus generated using the conventional packaging system is not feasible for transient expression of sgRNA and delivery of donor DNA for HDR due to the frequent integration of virus-derived DNA into the host genome following infection. To overcome this challenge, we leveraged the non-integrating nature of IDLV, due to a lack of a functional integrase enzyme, and combined it with our single-plasmid system to develop a novel approach for generating reporter knock-in cells by CRISPR-Cas9 genome editing. Remarkably, we found that the IDLV-based approach enables the generation of homozygous knock-in reporter cells with a similar efficiency to the single-plasmid electroporation. Despite optimised plasmid design and the incorporation of a constitutively expressed fluorescent reporter to minimise cells with random integration, PCR genotyping still revealed instances of unintended donor plasmid integration into the genome. While this may not significantly impact the utility of the established knock-in reporter cells for many applications, the cell lines generated using the IDLV approach exhibited minimal or undetectable levels of random integration. In line with this, ddPCR analysis confirms no alternation in the copy number at the target locus across all the approaches, and demonstrates a consistent 1:1 copy number ratio between the reporter and the *TSPAN8* gene, strongly suggesting that random integration via the IDLV method is negligible. We speculate that this is likely due to the substantially lower amount of donor DNA delivered into cells when using the IDLV system, as compared to the high DNA load introduced by electroporation.

Our approaches entirely rely on FACS sorting according to the expression levels of the correctly integrated knock-in fluorescent reporter driven by the endogenous promoter of the target gene. Hence, they are not suitable for generating knock-in cells expressing non-fluorescent protein reporters, such as LacZ, luciferases, or other enzymes. Compared to clonal fluorescent knock-in reporter lines established by previous strategies, homozygous knock-in cell pool lines engineered by our methods significantly eliminate the issue associated with inherited clonal variations within the parental cells. However, our approaches require the target gene’s endogenous expression to be detectable and sufficiently uniform. Accordingly, one key limitation is that the methods developed here are not feasible for generating reporter knock-in cells for the genes that are silent in the parent cells. While the design of the donor DNA and use of the IDLV-based delivery system profoundly reduce random integration and background expression of the knock-in reporter, this study does not address issues related to the off-target effect of CRISPR-Cas9 genome editing. Alternative CRISPR-Cas9 genome editing approaches are steadily advancing. One effective method involves electroporating Cas9 ribonucleoprotein (RNP) complexes along with donor plasmids, which has demonstrated high editing efficiency [30]. Another strategy employs adeno-associated virus (AAV) vectors to deliver single-stranded DNA templates [31]. The approaches developed in this study offer an additional, valuable and distinctive option. Finally, it is important to note that our study is dependent on HDR for knock-in efficiency, which can vary across different cell types and phases of the cell cycle, and some cells may preferentially utilise NHEJ, limiting the generalizability of our method in settings where HDR is less active, such as cells with a defect in BRCA1 function.

## 5. Conclusions

In summary, both the dual-plasmid and single-plasmid electroporation approaches, as well as the IDLV strategy, have proven to be rapid and effective methods for establishing homozygous fluorescent knock-in reporter cell pools, particularly when FACS sorting is used to enrich cells with high reporter expression. These methods together offer flexibility for the creation of homozygous gene-edited cell populations with minimal technical hurdles. Furthermore, we demonstrated the compatibility and utility of our reporter knock-in cell lines by performing a genome-wide CRISPR-Cas9 screen in TSPAN8-eGFP reporter cells, identifying several key regulators of *TSPAN8* transcription, including known transcription factors like SOX9 and novel candidates such as NF2 and DYRK1A. The findings from our screen offer new insights into the transcriptional regulation of *TSPAN8* in liver cancer and may have broader implications for understanding its role in cancer progression. Together, these methods provide a flexible toolkit for rapid generation of genetically uniform reporter cell pools. By combining rigorous validation with biological discovery, our work advances both genome-engineering technology and understanding of cancer gene regulation, with a dual impact characteristic of the methodological breakthroughs.

## Data Availability

Data supporting this study are available from the lead contact upon request. This paper does not include original code. Additional information needed to reanalyse the data can also be obtained from the lead contact upon request.

## Supplementary Materials

The following supporting information can be downloaded at: https://www.mdpi.com/article/10.3390/cells14151165/s1, Figure S1: Generation and validation of homozygous MEC TSPAN8-GFP knock-in reporter cells; Figure S2: Generation of JHH5 TSPAN8-GFP reporter cells by the single plasmid system and further characterization of MEC TSPAN8-GFP reporter cells; Figure S3: Long-range PCR and ddPCR validation of homozygous TSPAN8-GFP knock-in cell pools; Table S1: Resources and reagents; Table S2: sgRNAs used in this study; Table S3: PCR primers used in this study; Table S4: Candidate target genes with *p* Score < 0.01 from the CRISPR-Cas9 screen on TSPAN8-T2A-GFP JHH5 reporter cells.
