# Supplementary material for: Rapid and Robust Generation of Homozygous Fluorescent Reporter Knock-In Cell Pools by CRISPR-Cas9
Source: Cells. 2025 Jul 29;14(15):1165. doi: 10.3390/cells14151165 (PMC12346671; doi:10.3390/cells14151165)
Supplement: Supplementary file 1 [file cells-14-01165-s001.zip › Supplementary Figures.pdf]

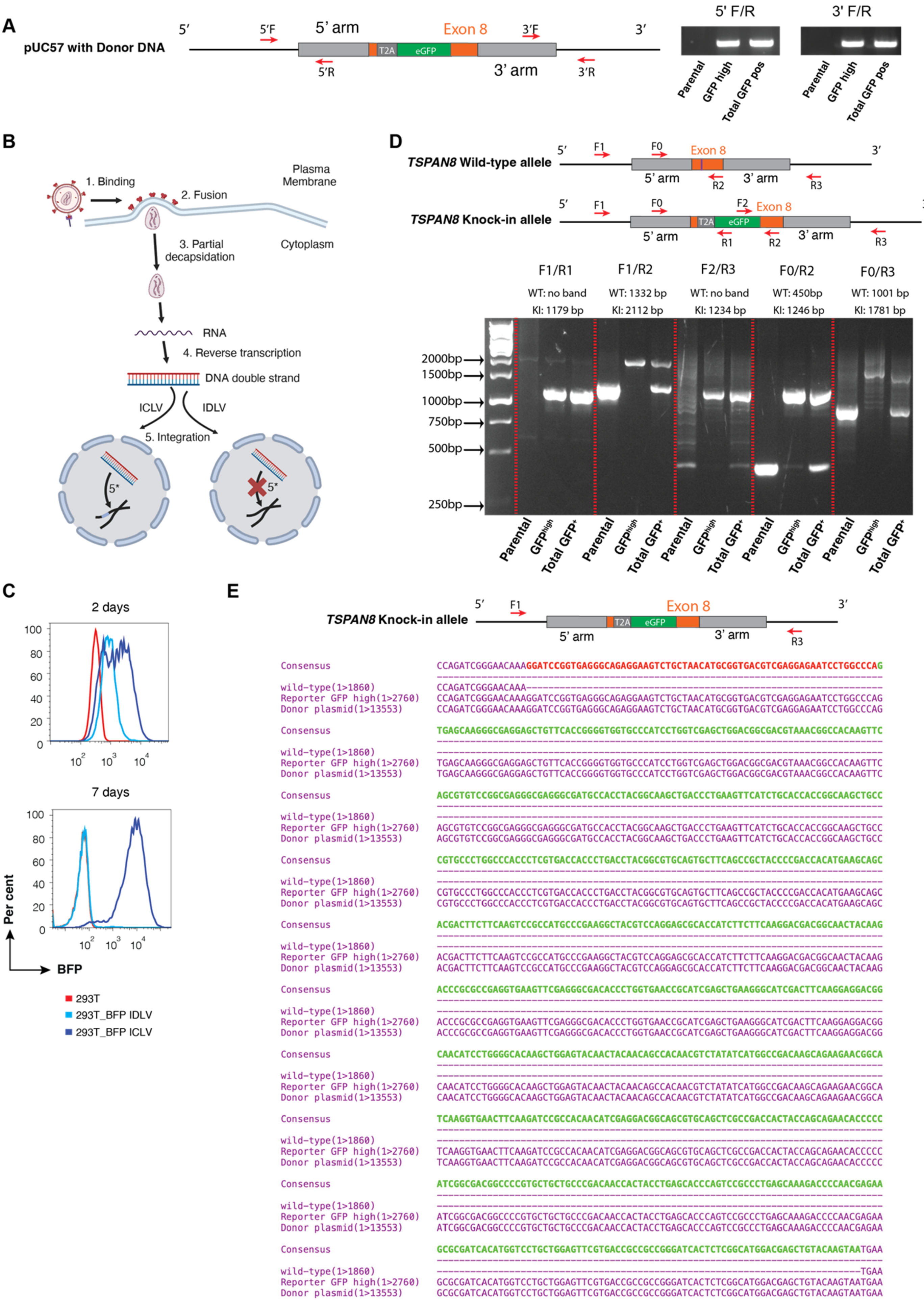
A
pUC57 with Donor DNA
5′
5'F
5' arm
Exon 8
3'F
3′
T2A
eGFP
5'R
3' arm
3'R
5' F/R
3' F/R
Parental
GFP high
Total GFP pos
B
1. Binding
2. Fusion
Plasma Membrane
3. Partial decapsidation
Cytoplasm
RNA
4. Reverse transcription
DNA double strand
ICLV
IDLV
5. Integration
C
2 days
7 days
Per cent
BFP
293T
293T_BFP IDLV
293T_BFP ICLV
D
TSPAN8 Wild-type allele
TSPAN8 Knock-in allele
F1
F0
F2
R1
R2
R3
F1/R1
F1/R2
F2/R3
F0/R2
F0/R3
WT: no band
KI: 1179 bp
WT: 1332 bp
KI: 2112 bp
WT: no band
KI: 1234 bp
WT: 450bp
KI: 1246 bp
WT: 1001 bp
KI: 1781 bp
2000bp
1500bp
1000bp
750bp
500bp
250bp
Parental
GFPhigh
Total GFP+
E
TSPAN8 Knock-in allele
Consensus
wild-type(1>1860)
Reporter GFP high(1>2760)
Donor plasmid(1>13553)

Figure S1. Generation and validation of homozygous MEC TSPAN8-GFP knock-in reporter cells.

A Detection of random integration in the established MEC GFP knock-in reporter cell pool generated by the dual plasmid system. PCR analysis was conducted with the indicated primers targeting the sequences spanning the pUC57 vector backbone and homology arms.

B Schematic representation of infection mechanisms for integrase-competent lentivirus (ICLV) and integrase-deficient lentivirus (IDLV). Both ICLV and IDLV bind to the cell membrane via viral envelope proteins and enter cells through receptor-mediated endocytosis. Upon entry, the viral RNA is reverse transcribed into cDNA in the cytoplasm. In the case of ICLV, the viral cDNA is actively transported into the nucleus, where it undergoes stable integration into the host genome via the integrase enzyme, leading to persistent gene expression. In contrast, IDLV carries a mutated integrase (D64E) and IDLV-derived cDNA remains episomal within the nucleus, allowing transient gene expression without permanent genomic integration or serving as donor DNA template for HDR. Created with BioRender.com.

C Validation of the transient expression feature of our sgRNA lentivirus packaged by the IDLV system. IDLV and ICLV viral preparations carrying a BFP-expressing sgRNA vector were used to infect cells under the identical condition. At 48 hours post-infection, BFP-positive infected cells by IDLV and ICLV was confirmed by FACS analysis, with ICLV-infected cells exhibiting stronger fluorescence intensity. By Day 7, BFP expression was nearly undetectable in IDLV-infected cells, whereas ICLV-infected cells remained BFP high.

D PCR genotyping of the established MEC reporter cell line generated by the IDLV system. The total GFP^{+} cells exhibited bands corresponding to both knock-in (KI) and wild-type (WT) alleles. The WT allele is absent in the reporter cell pool derived from the sorted GFP^{high} population.

E Sanger sequencing of the target region showing the successful and precise integration of the GFP reporter into the target genomic locus in the MEC TSPAN8-T2A-eGFP reporter cell pool. Sequencing was performed using primers located outside the 5′ and 3′ homology arms. Only the DNA sequence spanning the GFP reporter is shown.

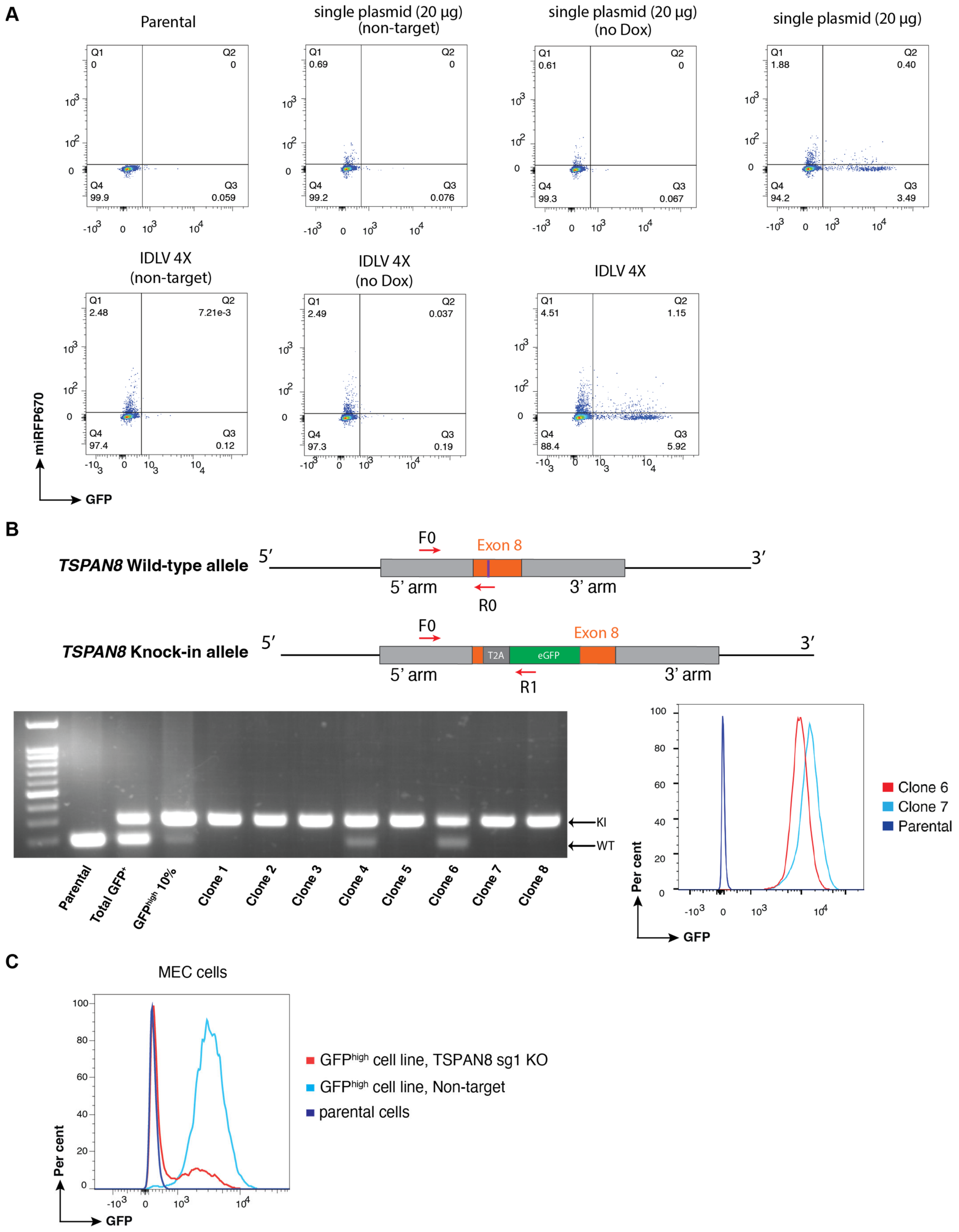
A
Parental
single plasmid (20 µg) (non-target)
single plasmid (20 µg) (no Dox)
single plasmid (20 µg)
Q1 0
Q2 0
Q4 99.9
Q3 0.059
Q1 0.69
Q2 0
Q4 99.2
Q3 0.076
Q1 0.61
Q2 0
Q4 99.3
Q3 0.067
Q1 1.88
Q2 0.40
Q4 94.2
Q3 3.49
IDLV 4X (non-target)
IDLV 4X (no Dox)
IDLV 4X
Q1 2.48
Q2 7.21e-3
Q4 97.4
Q3 0.12
Q1 2.49
Q2 0.037
Q4 97.3
Q3 0.19
Q1 4.51
Q2 1.15
Q4 88.4
Q3 5.92
miRFP670
GFP
B
TSPAN8 Wild-type allele
5′
3′
F0
Exon 8
5' arm
3' arm
R0
TSPAN8 Knock-in allele
T2A
eGFP
R1
KI
WT
Parental
Total GFP+
GFPhigh 10%
Clone 1
Clone 2
Clone 3
Clone 4
Clone 5
Clone 6
Clone 7
Clone 8
Clone 6
Clone 7
Parental
Per cent
GFP
C
MEC cells
GFPhigh cell line, TSPAN8 sg1 KO
GFPhigh cell line, Non-target
parental cells
Per cent
GFP

Figure S2. Generation of JHH5 TSPAN8-GFP reporter cells by the single plasmid system and further characterization of MEC TSPAN8-GFP reporter cells.

A. FACS analysis of JHH5 cells transfected with a single plasmid or infected with IDLV. The integration of knock-in reporter indicated by GFP expression is only detected in the cells treated with doxycycline, suggesting that induced expression of sgRNA is necessary for HDR and its tight control by the doxycycline system.

B. Clonal analysis of the MEC GFP^{high} cells generated by the dual-plasmid system. The top ~10% GFP^{high} cell population were isolated by FACS from cells directly electroporated with the dual-plasmid system. Single-cell clones were subsequently derived from this sorted population. PCR genotyping revealed that 6 out of 8 clones carried homozygous knock-in (KI/KI). FACS analysis confirmed that KI/KI clones displayed higher GFP expression levels than KI/+ cells.

C. Representative FACS plots showing the downregulation of GFP expression by TSPAN8 sgRNAs in the majority of cells in the MEC *TSPAN8-T2A-eGFP* pool established by the dual-plasmid system.

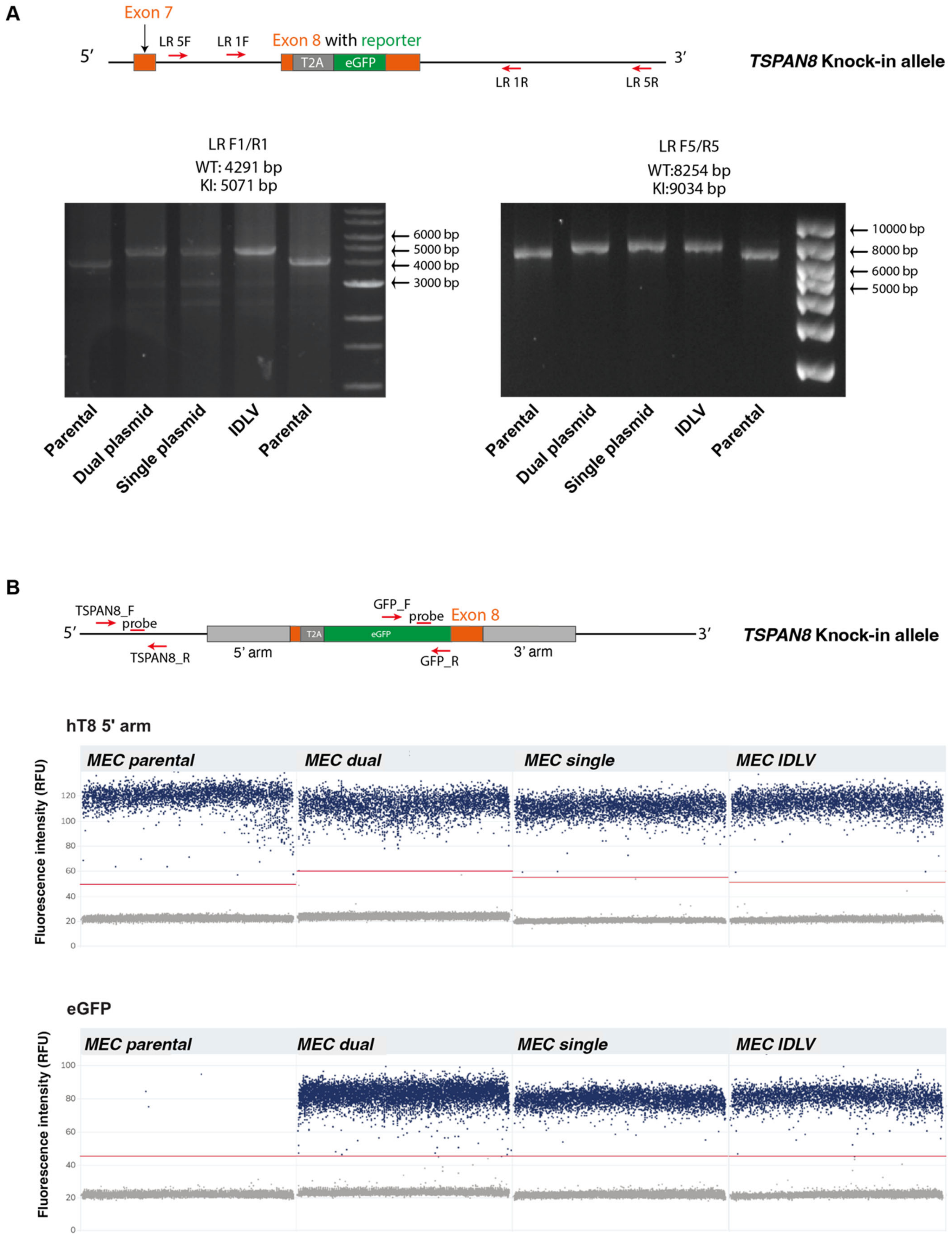
A
Exon 7
LR 5F
LR 1F
Exon 8 with reporter
5′
T2A
eGFP
3′
TSPAN8 Knock-in allele
LR 1R
LR 5R
LR F1/R1
WT: 4291 bp
KI: 5071 bp
6000 bp
5000 bp
4000 bp
3000 bp
LR F5/R5
WT:8254 bp
KI:9034 bp
10000 bp
8000 bp
6000 bp
5000 bp
Parental
Dual plasmid
Single plasmid
IDLV
Parental
Parental
Dual plasmid
Single plasmid
IDLV
Parental
B
TSPAN8_F
probe
TSPAN8_R
5′
5′ arm
T2A
eGFP
GFP_F
probe
Exon 8
GFP_R
3′ arm
3′
TSPAN8 Knock-in allele
hT8 5' arm
MEC parental
MEC dual
MEC single
MEC IDLV
Fluorescence intensity (RFU)
0
20
40
60
80
100
120
eGFP
MEC parental
MEC dual
MEC single
MEC IDLV
Fluorescence intensity (RFU)
0
20
40
60
80
100

Figure S3. Long-range PCR and ddPCR validation of homozygous TSPAN8-GFP knock-in cell pools.

A. Long-range PCR was performed on genomic DNA isolated from four MEC cell lines: the parental wild-type (unmodified) and three polyclonal reporter lines derived from GFP^{high} cells using electroporation of dual plasmids and single plasmid, and IDLV, respectively.

B. Droplet digital PCR (ddPCR) was performed to quantify the genomic copy numbers of GFP and endogenous TSPAN8 by using primers located upstream of the 5′ homology arm and within the GFP insert. A representative ddPCR plot is shown; each sample was analyzed in triplicate.
