# Supplementary material for: Rapid and Robust Generation of Homozygous Fluorescent Reporter Knock-In Cell Pools by CRISPR-Cas9
Source: Cells. 2025 Jul 29;14(15):1165. doi: 10.3390/cells14151165 (PMC12346671; doi:10.3390/cells14151165)
Supplement: Supplementary file 1 [file cells-14-01165-s001.zip › Table S1.pdf]

REAGENT or RESOURCE	SOURCE	IDENTIFIER
Antibodies		
Unconjugated rat monoclonal anti-human TSPAN8	R&D Systems	Clone 458811; Cat No. MAB4734
APC-conjugated anti-human TSPAN8, REAfinity™	Miltenyi Biotec	Clone REA443; Cat No. 130-106-811
Mouse monoclonal anti-human TSPAN8	A kind gift from Dr. Claude Boucheix, Inserm/University Paris Sud UA09	Clone Ts29.2, IgG2b
Plasmids Vectors for subcloning		
FgH1tUTG	A gift from Dr. Marco Herold at WEHI	Addgene (Plasmid No. 70183). It is used for doxycycline-inducing expression of sgRNA for CRISPR/Cas9 knockout in this study.
FgH1tUTB	In house	It was engineered by replacing eGFP in FgH1UTG with TagBFP. It is used for doxycycline-inducing expression of sgRNA for CRISPR/Cas9 knockout in this study.
FgH1tUT_miRFP670	In house	It was engineered by replacing eGFP in FgH1UTG with miRFP670. It is used for doxycycline-inducing expression of sgRNA for CRISPR/Cas9 knockout in this study.
FgH1tUT_miRFP670 with multiple cloning site (MCS)	In house	It was engineered to insert a multiple cloning site (MCS) into the sgRNA vector, enabling the efficient insertion of the donor DNA template. This modification facilitates the construction of a single plasmid carrying both the sgRNA expression cassette and the homology-directed repair (HDR) donor template.
pUC57_TSPAN8-T2A-GFP-SV40 polyA	Purchased from Genscript	The company synthesized the TSPAN8-T2A-GFP-SV40 polyA cassette and cloned it into the pUC57 vector. It’s used for making the KO reporter cells.
pUC57_TSPAN8-T2A-eGFP	Purchased from Genscript	The company synthesized the TSPAN8-T2A-eGFP cassette and cloned it into the pUC57 vector.

Single Plasmid vector: FgH1tUT_miRFP670 (MCS) with TSPAN8 sgRNA4 and the template donor TSPAN8-T2A-eGFP	In house	The TSPAN8 sgRNA4 was cloned into the vector as described in the Methods section. Subsequently, the TSPAN8-T2A-eGFP cassette was excised from the pUC57_TSPAN8-T2A-eGFP plasmid and inserted into the MCS site of the same vector.
Lentivirus package plasmids		
pMD2.G	Addgene	Addgene, Plasmid No. 12259
pRSV-Rev	Addgene	Addgene, Plasmid No. 12253
pMDLg/pRRE	Addgene	Addgene, Plasmid No. 12251
pBK43 (psPAX2-D64E)	A gift from Dr. Boris Kantor, Duke University- School of Medicine	integrase-deficient packaging cassette, DOI: 10.3791/56915
Lentivirus overexpression plasmids		
pFU_Cas9-T2A-mCherry	In house	Expression of Cas9 and mCherry
Experimental models: Cell lines		
MEC	CCR, Tohoku University	TKG 0629
JHH5	JCRB	JCRB1029
SNU878	KCLB	KCLB# 00878
HEK 293	ATCC	CRL-1573
Other reagents		
Puromycin Dihydrochloride	Goldbio	CAS No. 58-58-2; Cat No. P-600-100
4',6-Diamidino-2-phenylindole dihydrochloride (DAPI)	Invitrogen	CAS No. 28718-90-3; Cat No. D9542;
Deoxyribonuclease I (DNAse I)	Worthington Biochemical Corp	CAS No. 9003-98-9; Cat No. LS002140
Fetal Bovine Serum	Sigma Aldrich	Cat No. 12003C
IgG from rat serum	Sigma Aldrich	Cat No. I4131
SYBR™ Green PCR Master Mix	Thermo Fisher Scientific	Cat No. 4309155
TritonTM X-100	Sigma	Cat No. T8787
Horse serum	Thermo Fisher Scientific	Cat No. 16050130
DMEM media	Hyclone, Cytiva	Cat No. SH30022.01
fetal bovine serum	Sigma-Aldrich	Cat No. F9665
penicillin/streptomycin	Hyclone, Cytiva	Cat No. SV30010
William's E media	Pan Biotech	Cat No. P04-29510
Trypsin-EDTA (1X) solution	Thermo Fisher Scientific	Cat No. 25200-056
Pfu DNA polymerase	Promega	Cat No. M7745
Opti-MEM	Gibco™	Cat No.31985047
Polyethylenimine	Polysciences	Cat No. 23966-1

7-AAD	Caymanchem	CAS No. 7240-37-1; Cat No. 11397
One-4-All Genomic DNA Miniprep Kit	Bio Basic	Cat No. BS88504
protease and phosphatase inhibitor cocktail	Roche, Mannheim, Germany	Cat No. C762Q77
Gibco™ Trypsin-EDTA	Life Technologies Corporation	Cat No. 25200-056
1x non-enzymatic cell dissociation solution	Merck	MDL# MFCD00282844, Cat No. C5914
Software		
Image J	Fiji	RRID: SCR_003070
Gene information	NCBI	ftp://ftp.ncbi.nlm.nih.gov
Cancer Cell Line Encyclopedia (CCLE)	The Broad Institute of MIT & Harvard	https://portals.broadinstitute.org/ccle
Leica Application Suite X	Leica Microsystems GmbH	RRID:SCR_013673
GraphPad Prism 7	GraphPad Software	RRID: SCR_002798
Flowjo	Tree Star	RRID: SCR_008520
Others		
Neon™ NxT Electroporation System 100-μL Kit	Thermo Fisher Scientific	N10025
GoScriptTM Reverse Transcription Kit	Promega	Cat No. A5000
RNeasy Micro Kit	Qiagen	Cat No. 74004
