# Supplementary material for: Rapid and Robust Generation of Homozygous Fluorescent Reporter Knock-In Cell Pools by CRISPR-Cas9
Source: Cells. 2025 Jul 29;14(15):1165. doi: 10.3390/cells14151165 (PMC12346671; doi:10.3390/cells14151165)
Supplement: Supplementary file 1 [file cells-14-01165-s001.zip › Table S2.pdf]

Table S2

Target gene	sgRNA detail	Sequence of sgRNA	From the Sabatini sgRNA library or not	Used for generation of cell lines
h-TSPAN8	sgRNA1	GTACCCATATT GCTAATGCT	Not	MEC TSPAN8 sgRNA KO (GFP as reporter)
	sgRNA target the sequence within exon 2			JHH5 TSPAN8 sgRNA KO (GFP as reporter)
h-TSPAN8	sgRNA4	GATCGGGAAC AAATGAATCT	Not	MEC TSPAN8-T2A-eGFP (eGFP as reporter)
	sgRNA target the sequence near stop codon			JHH5 TSPAN8-T2A-eGFP (eGFP as reporter)
h-NF2	sgRNA4	CGTCACCATGG ACGCCGAGA	Yes	JHH5 TSPAN8-T2A-eGFP (eGFP as reporter) NF2 sg4 KO
				SNU878 NF2 sg4 KO
h-NF2	sgRNA9	TCTTTGAGCCT ACCTTGGCC	Yes	JHH5 TSPAN8-T2A-eGFP (eGFP as reporter) NF2 sg9 KO
				SNU878 NF2 sg9 KO
h-DYRK1A	sgRNA3	TCATTGGCACC ACTGAACAG	Yes	JHH5 TSPAN8-T2A-eGFP (eGFP as reporter) DYRK1A sg3 KO
				SNU878 DYRK1A sg3 KO
h-DYRK1A	sgRNA10	TTCAACCAAAA TACACCCGA	Yes	JHH5 TSPAN8-T2A-eGFP (eGFP as reporter) DYRK1A sg10 KO
				SNU878 DYRK1A sg10 KO
h-SOX9	sgRNA7	CCAGGAGAAC ACGTTCCCCA	Yes	JHH5 TSPAN8-T2A-eGFP (eGFP as reporter) SOX9 sg7 KO
				SNU878 SOX9 sg7 KO
h-SOX9	sgRNA8	GCCGTTGACGC GCACCGGCA	Yes	JHH5 TSPAN8-T2A-eGFP (eGFP as reporter) SOX9 sg8 KO
				SNU878 SOX9 sg8 KO
h-TSPAN8	sgRNA used to show the effect of TSPAN8-targeting sgRNAs on reporter expression	sgRNA1: GTACCCATATT GCTAATGCT<br><br>sgRNA2: CAATATGTCCA CAGCAACGT	Not	
