# Supplementary material for: Rapid and Robust Generation of Homozygous Fluorescent Reporter Knock-In Cell Pools by CRISPR-Cas9
Source: Cells. 2025 Jul 29;14(15):1165. doi: 10.3390/cells14151165 (PMC12346671; doi:10.3390/cells14151165)
Supplement: Supplementary file 1 [file cells-14-01165-s001.zip › Table S3.pdf]

Table S3

primer ID	Sequence of primer
pUC57 TSPAN8 random 5 arm F	TGCAAGGCGATTAAGTTGGGTAAC
pUC57 TSPAN8 random 5 arm R	CCATCTCAAGTAGCTCTCCCAAGAA
pUC57 TSPAN8 random 3 arm F	CAACCATTGGTGATAAGACTGAATC
pUC57 TSPAN8 random 3 arm R	GTGTGGAATTGTGAGCGGATAA
FgH1tUT TSPAN8 random 5 arm F	TCGCTATGTGTTCTGGGAAATC
FgH1tUT TSPAN8 random 5 arm R	GGTGACTTGCATGCTGTATCT
FgH1tUT TSPAN8 random 3 arm F	CTCTGGTACCCATCTGGATCTT
FgH1tUT TSPAN8 random 3 arm R	CGCCGAGAAGGGACTACTTT
gt TSPAN8-T2A-eGFP reporter line F0	GATGAGTTAGATAAGATTGGAACTGTC
gt TSPAN8-T2A-eGFP reporter line R0	GCATCCACAGATTCATTTGTTCC
gt TSPAN8-T2A-eGFP reporter line F1	AAGGCTCAGGATCAAGACAAA
gt TSPAN8-T2A-eGFP reporter line F2	GACGACGGCAACTACAAGA
gt TSPAN8-T2A-eGFP reporter line R1	GTTTACGTCGCCGTCCAGCTCGAC
gt TSPAN8-T2A-eGFP reporter line R2	AGCACTGTAGCACCTGAATAC
gt TSPAN8-T2A-eGFP reporter line R3	CTGTCTCAGCTTCCCAAGTAG
