# Supplementary material for: Rapid and Robust Generation of Homozygous Fluorescent Reporter Knock-In Cell Pools by CRISPR-Cas9
Source: Cells. 2025 Jul 29;14(15):1165. doi: 10.3390/cells14151165 (PMC12346671; doi:10.3390/cells14151165)
Supplement: Supplementary file 1 [file cells-14-01165-s001.zip › Table S4.pdf]

Table S4

No.	Gene ID	Full gene name	P-value of positive selection
1	ATXN7L3	Ataxin-7-like protein 3	4.51E-13
2	TSPAN8	Tetraspanin-8	4.95E-12
3	NF2	Merlin	1.57E-10
4	DYRK1A	Dual specificity tyrosine-phosphorylation-regulated kinase 1A	8.54E-07
5	CREBBP	CREB-binding protein	1.24E-06
6	TARSL2	Threonine--tRNA ligase 2	1.80E-05
7	IRF2	Interferon regulatory factor 2	2.41E-05
8	RPS6KA3	Ribosomal protein S6 kinase alpha-3	2.72E-05
9	NOMO1	Nodal modulator 1	4.73E-05
10	IKBIP	IKBKB interacting protein.	4.78E-05
11	SAV1	Protein salvador homolog 1	5.71E-05
12	CBX2	Chromobox protein homolog 2	7.24E-05
13	ENPP1	Ectonucleotide pyrophosphatase/phosphodiesterase family member 1	9.57E-05
14	ZNRF2	E3 ubiquitin-protein ligase ZNRF2	9.80E-05
15	SOX9	Transcription factor SOX-9	0.00010535
16	CTU1	Cytoplasmic tRNA 2-thiolation protein 1	0.00010895
17	PEBP4	Phosphatidylethanolamine-binding protein 4	0.0001136
18	TAOK1	Serine/threonine-protein kinase TAO1	0.00011884
19	BCKDK	[3-methyl-2-oxobutanoate dehydrogenase [lipoamide]] kinase	0.00011971
20	AXIN2	Axin-2	0.00012668
21	KANK1	KN motif and ankyrin repeat domain-containing protein 1	0.0001284
22	TBL2	Transducin beta like 2.	0.00014113
23	EIF4E2	Eukaryotic translation initiation factor 4E type 2	0.00014113
24	MAP3K9	Mitogen-activated protein kinase kinase kinase 9	0.00014701
25	GPR56	Adhesion G-protein coupled receptor G1	0.00016286
26	NABP2	SOSS complex subunit B1	0.00016733
27	LYZ	Lysozyme C	0.00016733
28	ZNF268	Zinc finger protein 268	0.00016877
29	ZNF527	Zinc finger protein 527	0.00016893
30	PTPN12	Tyrosine-protein phosphatase non-receptor type 12	0.00017277
31	IFT80	Intraflagellar transport protein 80 homolog	0.00017917
32	HAND2	Heart- and neural crest derivatives-expressed protein 2	0.0001792
33	C1orf43	Uncharacterized protein C1orf43	0.00018611
34	ZNF256	Zinc finger protein 256	0.00019352
35	LRRC70	Leucine-rich repeat-containing protein 70	0.00019397
36	KCNK9	Potassium channel subfamily K member 9	0.00021621
37	EZR	Ezrin	0.00023879
38	TMEM30B	Cell cycle control protein 50B	0.00024655
39	TPPP3	Tubulin polymerization-promoting protein family member 3	0.00026546
40	PTPRE	Receptor-type tyrosine-protein phosphatase epsilon	0.00027108
41	OAS3	2'-5'-oligoadenylate synthase 3	0.00027245
42	C16orf58	RUS1 family protein C16orf58.	0.00027416
43	FMO4	Dimethylaniline monooxygenase [N-oxide-forming] 4	0.0002927
44	YPEL3	Protein yippee-like 3	0.00029283
45	TSSK1B	Testis-specific serine/threonine-protein kinase 1	0.00035292

Table S4

46	CLDN5	Claudin-5	0.00035421
47	CDC20B	Cell division cycle 20B	0.00038108
48	HOXB4	Homeobox protein Hox-B4	0.00038108
49	C15orf52	Coiled-coil domain containing 9B.	0.00038108
50	CRYBA1	Beta-crystallin A3	0.00039717
51	SEC24A	Protein transport protein Sec24A	0.00043935
52	HDAC2	Histone deacetylase 2	0.0004482
53	ITGAX	Integrin alpha-X	0.00044947
54	REP15	Rab15 effector protein	0.00045781
55	DEDD	Death effector domain-containing protein	0.00046912
56	SRA1	Steroid receptor RNA activator 1	0.00047029
57	WWC1	Protein KIBRA	0.00050065
58	PARP11	Protein mono-ADP-ribosyltransferase PARP11	0.00050244
59	SLC2A13	Proton myo-inositol cotransporter	0.0005046
60	C16orf96	Uncharacterized protein C16orf96	0.00050864
61	HFE	Hereditary hemochromatosis protein	0.00051275
62	NUDT4	Diphosphoinositol polyphosphate phosphohydrolase NUDT4B	0.00051775
63	S100A14	Protein S100-A14	0.00051926
64	USF1	Upstream stimulatory factor 1	0.00053134
65	TJAP1	Tight junction associated protein 1.	0.00054619
66	C17orf80	Uncharacterized protein C17orf80	0.00055083
67	ITGA9	Integrin alpha-9	0.00057306
68	TAF7	Transcription initiation factor TFIID subunit 7	0.00059618
69	AAED1	Peroxiredoxin-like 2C	0.00060262
70	RHD	Rh blood group D antigen	0.00061227
71	MGRN1	E3 ubiquitin-protein ligase MGRN1	0.00061315
72	IQCD	Dynein regulatory complex protein 10	0.00061315
73	FAXC	Failed axon connections homolog	0.00062346
74	HMOX1	Heme oxygenase 1	0.00064501
75	KRTAP19-4	Keratin-associated protein 19-4	0.00066689
76	RAI2	Retinoic acid induced 2.	0.0006675
77	CASC3	Protein CASC3	0.00069367
78	ARL13B	ADP-ribosylation factor-like protein 13B	0.00070836
79	ZNF77	Zinc finger protein 77	0.00072507
80	CCL24	C-C motif chemokine 24	0.00073148
81	PDPK1	3-phosphoinositide-dependent protein kinase 1	0.00073287
82	SCYL2	SCY1-like protein 2	0.00073682
83	MEP1A	Meprin A subunit alpha.	0.00074223
84	SRL	Sarcalumenin	0.00076079
85	SMARCAD1	ATP-dependent helicase 1	0.00079665
86	PTCHD1	Patched domain-containing protein 1	0.00080756
87	WBSCR17	Polypeptide N-acetylgalactosaminyltransferase 17	0.00081638
88	EDEM1	ER degradation-enhancing alpha-mannosidase-like protein 1	0.00081856
89	C14orf1	Ergosterol biosynthesis 28 homolog	0.00083661
90	CLN6	CLN6 transmembrane ER protein.	0.00085883
91	ZNF416	Zinc finger protein 416	0.00086427
92	MGAT2	Alpha-1	0.00087904
93	SLC35A5	Probable UDP-sugar transporter protein SLC35A5	0.00089265
94	HOXC4	Homeobox protein Hox-C4	0.00092702

Table S4

**95**	CHML	Rab proteins geranylgeranyltransferase component A 2	0.00093217
**96**	TMEM168	Transmembrane protein 168	0.00093485
**97**	NKD1	Protein naked cuticle homolog 1	0.00097131
**98**	MGMT	Methylated-DNA--protein-cysteine methyltransferase	0.00098561
**99**	TPSG1	Tryptase gamma 1.	0.00098912
**100**	IL4I1	L-amino-acid oxidase	0.0009979
**101**	DMTF1	Cyclin-D-binding Myb-like transcription factor 1	0.0010362
**102**	MIER3	Mesoderm induction early response protein 3	0.0010587
**103**	CDA	Cytidine deaminase	0.0011238
**104**	F11R	Junctional adhesion molecule A	0.0011326
**105**	LRRFIP2	Leucine-rich repeat flightless-interacting protein 2	0.0011326
**106**	PDCD10	Programmed cell death protein 10	0.0011531
**107**	FNBP1L	Formin-binding protein 1-like	0.0011584
**108**	PRDM14	PR domain zinc finger protein 14	0.00116
**109**	ARHGEF18	Rho/Rac guanine nucleotide exchange factor 18.	0.0011624
**110**	BLOC1S5	Biogenesis of lysosome-related organelles complex 1 subunit 5	0.0011648
**111**	SPANXN4	Sperm protein associated with the nucleus on the X chromosome N4	0.001176
**112**	PRNP	Major prion protein	0.001237
**113**	SSFA2	Protein ITPRID2	0.0012404
**114**	HOXC13	Homeobox protein Hox-C13	0.0012405
**115**	REV1	DNA repair protein REV1	0.0012922
**116**	SORBS3	Vinexin	0.0012965
**117**	IQCH	IQ domain-containing protein H	0.0013053
**118**	CCDC122	Coiled-coil domain containing 122.	0.0013297
**119**	RAB21	Ras-related protein Rab-21	0.0013589
**120**	AQP5	Aquaporin-5	0.0013654
**121**	MTM1	Myotubularin	0.0013694
**122**	SERPINA7	Thyroxine-binding globulin	0.0013937
**123**	DHRS4L2	Dehydrogenase/reductase SDR family member 4-like 2	0.001417
**124**	UBE2G1	Ubiquitin-conjugating enzyme E2 G1	0.0014254
**125**	ZNF600	Zinc finger protein 600	0.0014268
**126**	ABCB11	Bile salt export pump	0.0014301
**127**	ARMC2	Armadillo repeat-containing protein 2	0.0014381
**128**	LRRC19	Leucine-rich repeat-containing protein 19	0.0014414
**129**	GALNS	N-acetylgalactosamine-6-sulfatase	0.0014451
**130**	MED17	Mediator of RNA polymerase II transcription subunit 17	0.0014583
**131**	INVS	Inversin	0.0014924
**132**	RWDD3	RWD domain-containing protein 3	0.0014944
**133**	ABCF3	ATP-binding cassette sub-family F member 3	0.0015376
**134**	PPP1R36	Protein phosphatase 1 regulatory subunit 36	0.0015393
**135**	C1orf192	Protein Flattop	0.0015634
**136**	TCL1B	T-cell leukemia/lymphoma protein 1B	0.0015727
**137**	G3BP2	Ras GTPase-activating protein-binding protein 2	0.0015735
**138**	PPFIBP1	Liprin-beta-1	0.0015832
**139**	ARF3	ADP-ribosylation factor 3	0.0015946
**140**	ADIPOR1	Adiponectin receptor protein 1	0.0016008
**141**	LTBP1	Latent-transforming growth factor beta-binding protein 1	0.0016056
**142**	UNC119	Protein unc-119 homolog A	0.0016746
**143**	FDCSP	Follicular dendritic cell secreted peptide	0.0017093

Table S4

144	ERO1L	ERO1-like protein alpha	0.0017183
145	CCDC114	Coiled-coil domain-containing protein 114	0.0017265
146	P2RX4	P2X purinoceptor 4	0.0017657
147	WDR90	WD repeat-containing protein 90	0.0018087
148	VEZF1	Vascular endothelial zinc finger 1	0.0018367
149	KLHL21	Kelch-like protein 21	0.0018765
150	KIAA0907	KH homology domain-containing protein 4	0.0018765
151	PIK3R3	Phosphatidylinositol 3-kinase regulatory subunit gamma	0.0018765
152	GUCD1	Protein GUCD1	0.0019175
153	PLEKHF2	Pleckstrin homology domain-containing family F member 2	0.0019175
154	ASTE1	Protein asteroid homolog 1	0.0019175
155	WSCD2	WSC domain containing 2	0.0019175
156	OTX2	Homeobox protein OTX2	0.0019593
157	DKK2.00	Dickkopf-related protein 2	0.0020195
158	ELL2	RNA polymerase II elongation factor ELL2	0.0020608
159	C5orf48	Testis-expressed protein 43	0.0020797
160	MXRA7	Matrix-remodeling-associated protein 7	0.0020888
161	SHE	SH2 domain-containing adapter protein E	0.0020978
162	FKBP5	Peptidyl-prolyl cis-trans isomerase FKBP5	0.0021217
163	C7orf76	Putative protein SEM1	0.0021336
164	OSER1	Oxidative stress-responsive serine-rich protein 1	0.0021399
165	LARP4	La-related protein 4	0.002143
166	NSD1	Histone-lysine N-methyltransferase	0.0021452
167	TSACC	TSSK6-activating co-chaperone protein	0.0021588
168	HAPLN4	Hyaluronan and proteoglycan link protein 4	0.0021856
169	UMODL1	Uromodulin like 1.	0.0022002
170	TPD52L2	Tumor protein D54	0.0022139
171	ZMYM6NB	Transmembrane protein 35B.	0.0022424
172	FCER1A	High affinity immunoglobulin epsilon receptor subunit alpha	0.0022514
173	BTBD7	BTB/POZ domain-containing protein 7	0.0022604
174	TAS2R9	Taste receptor type 2 member 9	0.0022609
175	MLYCD	Malonyl-CoA decarboxylase	0.0022972
176	CD1E	T-cell surface glycoprotein CD1e	0.0023012
177	SORD	Sorbitol dehydrogenase	0.0023014
178	CD38	ADP-ribosyl cyclase/cyclic ADP-ribose hydrolase 1	0.0023051
179	FGF14	Fibroblast growth factor 14	0.0023201
180	C11orf54	Ester hydrolase C11orf54	0.0023323
181	KRTAP9-6	Keratin-associated protein 9-6	0.0023433
182	PAPD4	Poly(A) RNA polymerase GLD2	0.0023501
183	CENPH	Centromere protein H	0.0023568
184	KCNIP4	Kv channel-interacting protein 4	0.0023755
185	TCN2	Transcobalamin-2	0.0023842
186	TINAGL1	Tubulointerstitial nephritis antigen-like	0.0024049
187	PRDM12	PR domain zinc finger protein 12	0.0024196
188	FMN1	Formin-1	0.002441
189	APOBEC3A	DNA dC->dU-editing enzyme APOBEC-3A	0.0024681
190	MPV17L	Mpv17-like protein	0.0024804
191	ZNRF3	E3 ubiquitin-protein ligase ZNRF3	0.0025129
192	INS	Insulin A chain	0.0025229

Table S4

193	DHDH	Dihydrodiol dehydrogenase	0.0025482
194	C1orf101	Cation channel sperm-associated protein subunit epsilon	0.0025614
195	HGSNAT	Heparan-alpha-glucosaminide N-acetyltransferase	0.0025667
196	CD63	CD63 antigen	0.0025765
197	GOT1	Aspartate aminotransferase	0.0026055
198	PTGES3L	Putative protein PTGES3L	0.0026902
199	APOBEC3H	DNA dC->dU-editing enzyme APOBEC-3H	0.0026991
200	TMEM63B	CSC1-like protein 2	0.0027346
201	HIST1H1D	Histone H1.3	0.0027346
202	PCDHB5	Protocadherin beta-5	0.0027346
203	CTSS	Cathepsin S	0.0027346
204	MIER1	Mesoderm induction early response protein 1	0.0027419
205	KCTD15	BTB/POZ domain-containing protein KCTD15	0.0027827
206	VIM	Vimentin	0.0028054
207	CDHR2	Cadherin-related family member 2	0.0028161
208	ADAMTS4	A disintegrin and metalloproteinase with thrombospondin motifs 4	0.0028526
209	ZNF586	Zinc finger protein 586	0.0028685
210	DHX29	ATP-dependent RNA helicase DHX29	0.0028806
211	ACSL6	Long-chain-fatty-acid--CoA ligase 6	0.0028932
212	ZNF107	Zinc finger protein 107.	0.0028993
213	CAST	Calpastatin	0.0029078
214	POTEC	POTE ankyrin domain family member C.	0.0029217
215	POLR1D	DNA-directed RNA polymerases I and III subunit RPAC2	0.0029526
216	SLC5A4	Solute carrier family 5 member 4	0.0029612
217	DSCC1	Sister chromatid cohesion protein DCC1	0.0029631
218	PCDHGA6	Protocadherin gamma-A6	0.0029631
219	FBXO6	F-box only protein 6	0.0029631
220	SOX8	Transcription factor SOX-8	0.0029631
221	ZNF565	Zinc finger protein 565	0.0029876
222	MDFIC	MyoD family inhibitor domain-containing protein	0.0029892
223	C4B	Complement C4-B alpha chain	0.0029992
224	MYH14	Myosin-14	0.0030314
225	ZFR	Zinc finger RNA-binding protein	0.0030429
226	CELF2	CUGBP Elav-like family member 2	0.003064
227	DNALI1	Axonemal dynein light intermediate polypeptide 1	0.0031031
228	C4orf26	Odontogenesis associated phosphoprotein	0.0031168
229	SYTL4	Synaptotagmin-like protein 4	0.0031168
230	USP22	Ubiquitin carboxyl-terminal hydrolase 22	0.0031168
231	SKIDA1	SKI/DACH domain containing 1	0.0031364
232	FAM73A	Mitoguardin 1	0.0031365
233	CARNS1	Carnosine synthase 1	0.0031632
234	WDR66	Cilia- and flagella-associated protein 251	0.0031723
235	MYBPC1	Myosin-binding protein C	0.0032053
236	NUFIP2	Nuclear fragile X mental retardation-interacting protein 2	0.0032234
237	XYLT1	Xylosyltransferase 1	0.0032367
238	ITGB2	Integrin beta-2	0.0032585
239	PA2G4	Proliferation-associated protein 2G4	0.0032836
240	PGM1	Phosphoglucomutase-1	0.0032937
241	THNSL2	Threonine synthase-like 2	0.0032961
242	AJUBA	LIM domain-containing protein ajuba	0.0033348

Table S4

243	QSOX2	Sulfhydryl oxidase 2	0.0033415
244	FAM153B	Protein FAM153B	0.0033437
245	CYSLTR1	Cysteinyl leukotriene receptor 1	0.0033621
246	CXorf61	Kita-kyushu lung cancer antigen 1	0.0034005
247	GABARAPL1	Gamma-aminobutyric acid receptor-associated protein-like 1	0.003418
248	RASA1	Ras GTPase-activating protein 1	0.0034194
249	METTL15	Probable methyltransferase-like protein 15	0.003443
250	HNRNPA2B1	Heterogeneous nuclear ribonucleoproteins A2/B1	0.0034486
251	ALDH16A1	Aldehyde dehydrogenase 16 family member A1.	0.0034554
252	CXCL3	C-X-C motif chemokine 3	0.003501
253	C1orf52	UPF0690 protein C1orf52	0.0035211
254	CBR3	Carbonyl reductase [NADPH] 3	0.0035672
255	RORA	Nuclear receptor ROR-alpha	0.0035797
256	LYG1	Lysozyme g1.	0.0036067
257	TMED6	Transmembrane p24 trafficking protein 6.	0.0036445
258	JAM3	Junctional adhesion molecule C	0.0036534
259	ABAT	4-aminobutyrate aminotransferase	0.0036596
260	GLA	Galactosidase alpha	0.0036867
261	LSS	Lanosterol synthase	0.0036951
262	CELA3B	Chymotrypsin-like elastase family member 3B	0.0037137
263	GPRIN3	G protein-regulated inducer of neurite outgrowth 3	0.0037423
264	HRH1	Histamine H1 receptor	0.0037583
265	ZNF667	Zinc finger protein 667	0.0037592
266	LINGO4	Leucine-rich repeat and immunoglobulin-like domain-containing nogo receptor-interacting protein 4	0.0037624
267	RBM12B	RNA binding motif protein 12B.	0.003783
268	PPP2R3C	Serine/threonine-protein phosphatase 2A regulatory subunit B'' subunit gamma	0.0038271
269	PYHIN1	Pyrin and HIN domain-containing protein 1	0.003885
270	TNRC6B	Trinucleotide repeat-containing gene 6B protein	0.003899
271	SCYL3	Protein-associating with the carboxyl-terminal domain of ezrin	0.003904
272	PLXNA3	Plexin-A3	0.0039302
273	CLINT1	Clathrin interactor 1	0.0039305
274	PNPLA8	Calcium-independent phospholipase A2-gamma	0.0039451
275	PBLD	Phenazine biosynthesis like protein domain containing.	0.0039843
276	MSL1	Male-specific lethal 1 homolog	0.0039973
277	MEI1	Meiosis inhibitor protein 1	0.0040151
278	PGBD4	piggyBac transposable element derived 4.	0.0040395
279	MYLPF	Myosin light chain	0.0040639
280	LRP3	Low-density lipoprotein receptor-related protein 3	0.0041674
281	KIAA1024L	Major intrinsically disordered NOTCH2-binding receptor 1-like	0.0042457
282	CLTA	Clathrin light chain A	0.0042929
283	ZNF433	Zinc finger protein 433	0.0042929
284	SLC38A4	Sodium-coupled neutral amino acid transporter 4	0.0042929
285	ASPHD2	Aspartate beta-hydroxylase domain-containing protein 2	0.0042929
286	SENP1	Sentrin-specific protease 1	0.0042929
287	PTH2	Tuberoinfundibular peptide of 39 residues	0.0042929
288	CCDC135	Dynein regulatory complex subunit 7	0.0042929

Table S4

**289**	KBTBD2	Kelch repeat and BTB domain containing 2.	0.0042929
**290**	SH2D2A	SH2 domain-containing protein 2A	0.0043364
**291**	AFM	Afamin	0.0043364
**292**	DDX26B	Integrator complex subunit 6 like.	0.0043364
**293**	EPHX4	Epoxide hydrolase 4.	0.0043364
**294**	ANKRD36	Ankyrin repeat domain-containing protein 36A	0.0043364
**295**	TYR	Tyrosinase	0.0043364
**296**	AP4M1	AP-4 complex subunit mu-1	0.0043364
**297**	CASZ1	Zinc finger protein castor homolog 1	0.0044129
**298**	PPP1R15B	Protein phosphatase 1 regulatory subunit 15B	0.0044171
**299**	PTPN21	Protein tyrosine phosphatase non-receptor type 21.	0.0044431
**300**	SLC35D1	UDP-glucuronic acid/UDP-N-acetylgalactosamine transporter	0.0045692
**301**	CTF1	Cardiotrophin-1	0.0045768
**302**	MACROD2	ADP-ribose glycohydrolase MACROD2	0.0045938
**303**	ST7	Suppressor of tumorigenicity 7 protein	0.0046664
**304**	CCNB2	G2/mitotic-specific cyclin-B2	0.004708
**305**	LAMTOR1	Ragulator complex protein LAMTOR1	0.0047343
**306**	GHITM	Growth hormone-inducible transmembrane protein	0.0047398
**307**	ABHD11	Protein ABHD11	0.0047416
**308**	VGF	Neuroendocrine regulatory peptide-1	0.0047865
**309**	ZNF552	Zinc finger protein 552	0.004851
**310**	FOXRED2	FAD-dependent oxidoreductase domain-containing protein 2	0.0048908
**311**	PTN	Pleiotrophin	0.0049151
**312**	GALNT8	Probable polypeptide N-acetylgalactosaminyltransferase 8	0.0049668
**313**	XIRP1	Xin actin-binding repeat-containing protein 1	0.0049742
**314**	ACSM4	Acyl-coenzyme A synthetase ACSM4	0.005
**315**	ZNF205	Zinc finger protein 205	0.0050268
**316**	EP300	Histone acetyltransferase p300	0.0050318
**317**	AURKB	Aurora kinase B	0.005039
**318**	CADM2	Cell adhesion molecule 2	0.0050459
**319**	UCHL1	Ubiquitin carboxyl-terminal hydrolase isozyme L1	0.0050869
**320**	KLK9	Kallikrein-9	0.005141
**321**	ZKSCAN7	Zinc finger protein with KRAB and SCAN domains 7	0.0051469
**322**	COL1A1	Collagen alpha-1(I) chain	0.0051754
**323**	POLR2G	DNA-directed RNA polymerase II subunit RPB7	0.005207
**324**	MAGEB18	Melanoma-associated antigen B18	0.0052096
**325**	PRKCB	Protein kinase C beta type	0.0052227
**326**	BCORL1	BCL-6 corepressor-like protein 1	0.0052414
**327**	PRAF2	PRA1 family protein 2	0.0052552
**328**	DHRS7C	Dehydrogenase/reductase SDR family member 7C	0.0053271
**329**	YOD1	Ubiquitin thioesterase OTU1	0.0053301
**330**	CBFA2T3	Protein CBFA2T3	0.0053633
**331**	FGD6	FYVE	0.005385
**332**	UBE2L3	Ubiquitin conjugating enzyme E2 L3.	0.0053871
**333**	ATP6V0E2	V-type proton ATPase subunit e 2	0.005403
**334**	LEPRE1	Prolyl 3-hydroxylase 1	0.0054444
**335**	HOXB2	Homeobox protein Hox-B2	0.0054472
**336**	PRSS37	Probable inactive serine protease 37	0.0054719
**337**	SPRED2	Sprouty-related	0.0054737

Table S4

**338**	OFD1	Oral-facial-digital syndrome 1 protein	0.0054906
**339**	PAH	Phenylalanine-4-hydroxylase	0.0055072
**340**	CITED4	Cbp/p300-interacting transactivator 4	0.0055152
**341**	PTCHD2	Protein dispatched homolog 3	0.005526
**342**	PTPRZ1	Receptor-type tyrosine-protein phosphatase zeta	0.0055617
**343**	ABCD4	ATP-binding cassette sub-family D member 4	0.005587
**344**	WIPI1	WD repeat domain phosphoinositide-interacting protein 1	0.0055973
**345**	CD247	T-cell surface glycoprotein CD3 zeta chain	0.005604
**346**	KCNJ4	Inward rectifier potassium channel 4	0.0056386
**347**	MCU	Calcium uniporter protein	0.0056873
**348**	BGLAP	Osteocalcin	0.0057053
**349**	RPUSD2	RNA pseudouridine synthase domain containing 2.	0.0057053
**350**	GYPC	Glycophorin-C	0.0057053
**351**	NIN	Ninein	0.0057053
**352**	ARL3.00	ADP-ribosylation factor-like protein 3	0.0057147
**353**	CTXN1	Cortexin-1	0.0057474
**354**	SPA17	Sperm surface protein Sp17	0.0057624
**355**	ZNF17	Zinc finger protein 17	0.0058498
**356**	CRAT	Carnitine O-acetyltransferase	0.0058539
**357**	ZNF280A	Zinc finger protein 280A	0.0058942
**358**	LPPR5	Phospholipid phosphatase-related protein type 5	0.0059259
**359**	IFNB1	Interferon beta	0.0059306
**360**	RDH10	Retinol dehydrogenase 10	0.0059574
**361**	MED12L	Mediator of RNA polymerase II transcription subunit 12-like protein	0.0059586
**362**	IPO8	Importin-8	0.0060287
**363**	CYP2W1	Cytochrome P450 2W1	0.0060385
**364**	ALMS1	Alstrom syndrome protein 1	0.0060927
**365**	ZFP42	Zinc finger protein 42 homolog	0.0061379
**366**	SNX17	Sorting nexin-17	0.0061467
**367**	GMCL1	Germ cell-less protein-like 1	0.0062007
**368**	CDCA8	Borealin	0.0062194
**369**	MKNK2	MAP kinase-interacting serine/threonine-protein kinase 2	0.0062211
**370**	GRAPL	GRB2-related adapter protein-like	0.0062211
**371**	NRTN	Neurturin	0.0062211
**372**	WDR34	WD repeat-containing protein 34	0.0062211
**373**	HIST2H2BE	Histone H2B type 2-E	0.0062211
**374**	HBA1	Hemoglobin subunit alpha 1.	0.0062211
**375**	KLHL13	Kelch-like protein 13	0.0062211
**376**	STC1	Stanniocalcin-1	0.0062275
**377**	RAB3B	Ras-related protein Rab-3B	0.006249
**378**	IMPA2	Inositol monophosphatase 2	0.0062875
**379**	SPAG7	Sperm associated antigen 7.	0.0063324
**380**	CACUL1	CDK2-associated and cullin domain-containing protein 1	0.0063475
**381**	SMOX	Spermine oxidase	0.0063627
**382**	GPATCH11	G-patch domain containing 11.	0.0064054
**383**	LIMA1	LIM domain and actin-binding protein 1	0.0064074
**384**	BTF3L4	Basic transcription factor 3 like 4	0.006432
**385**	ZFHX2	Zinc finger homeobox protein 2	0.0064674
**386**	SOCS4	Suppressor of cytokine signaling 4	0.0064707
**387**	TMEM244	Transmembrane protein 244.	0.0065008

Table S4

388	ZNF441	Zinc finger protein 441	0.006517
389	OLFML2B	Olfactomedin-like protein 2B	0.0065274
390	HBZ	Hemoglobin subunit zeta	0.0065675
391	IL17D	Interleukin-17D	0.0065699
392	ATXN7L3B	Ataxin-7-like protein 3B	0.0066042
393	BEST3	Bestrophin-3	0.0066326
394	CCDC110	Coiled-coil domain containing 110.	0.0066474
395	SFTA3	Surfactant-associated protein 3	0.0067074
396	MTCH1	Mitochondrial carrier homolog 1	0.0067117
397	PDGFD	Platelet-derived growth factor D	0.006714
398	MZT2A	Mitotic spindle organizing protein 2A	0.0067336
399	AKAP5	A-kinase anchor protein 5	0.0067391
400	CT45A5	Cancer/testis antigen family 45 member A5.	0.0067406
401	NMRAL1	NmrA-like family domain-containing protein 1	0.0067803
402	IDS	Iduronate 2-sulfatase 14 kDa chain	0.0067807
403	CACNG2	Voltage-dependent calcium channel gamma-2 subunit	0.0068344
404	ABI1	Abl interactor 1	0.0068383
405	LDLRAD3	Low-density lipoprotein receptor class A domain-containing protein 3	0.0068439
406	AMER2	APC membrane recruitment protein 2	0.0068555
407	TRH	Pro-thyrotropin-releasing hormone	0.0069287
408	GKAP1	G kinase-anchoring protein 1	0.0069848
409	ZNF124	Zinc finger protein 124	0.0070018
410	NPSR1	Neuropeptide S receptor	0.0070072
411	PDILT	Protein disulfide-isomerase-like protein of the testis	0.0070867
412	C7orf41	Maturin	0.0070994
413	STK31	Serine/threonine kinase 31	0.0071133
414	MAGEC3	Melanoma-associated antigen C3	0.0071525
415	SETDB1	Histone-lysine N-methyltransferase SETDB1	0.007187
416	SPANXB1	Sperm protein associated with the nucleus on the X chromosome B1	0.0071914
417	SYF2	Pre-mRNA-splicing factor SYF2	0.0072163
418	LRRC37A2	Leucine-rich repeat-containing protein 37A2	0.0072178
419	ZRANB1	Ubiquitin thioesterase ZRANB1	0.0072631
420	PRDM4	PR domain zinc finger protein 4	0.0072631
421	SLC10A1	Sodium/bile acid cotransporter	0.0073258
422	BRD2	Bromodomain-containing protein 2	0.0073376
423	ME3	NADP-dependent malic enzyme	0.0073387
424	SIRT5	NAD-dependent protein deacylase sirtuin-5	0.007346
425	HIST1H4L	Histone H4	0.0073798
426	PEX6	Peroxisome assembly factor 2	0.0074268
427	ZNF429	Zinc finger protein 429	0.0074421
428	ARPC5	Actin-related protein 2/3 complex subunit 5	0.0075054
429	VNN2.00	Vascular non-inflammatory molecule 2	0.0075054
430	TFAP4	Transcription factor AP-4	0.0075467
431	BPIFC	BPI fold containing family C.	0.00755
432	UBE2H	Ubiquitin-conjugating enzyme E2 H	0.0076162
433	FAM19A2	Chemokine-like protein TAFA-2	0.0076714
434	CALCOCO1	Calcium-binding and coiled-coil domain-containing protein 1	0.0076732
435	RNF34	E3 ubiquitin-protein ligase RNF34	0.0077264

Table S4

**436**	CLEC2L	C-type lectin domain family 2 member L.	0.0077729
**437**	CLEC9A	C-type lectin domain family 9 member A	0.0077864
**438**	C5orf47	Uncharacterized protein C5orf47	0.0078196
**439**	CLDN15	Claudin-15	0.007865
**440**	PDE3B	cGMP-inhibited 3'	0.0078876
**441**	GALR1	Galanin receptor type 1	0.0078876
**442**	IL1RL2	Interleukin-1 receptor-like 2	0.0078876
**443**	SNN	Stannin	0.0078876
**444**	PCDHB12	Protocadherin beta-12	0.0078876
**445**	THUMPD3	THUMP domain containing 3.	0.0078876
**446**	IL7R	Interleukin-7 receptor subunit alpha	0.0078876
**447**	PDHX	Pyruvate dehydrogenase protein X component	0.0078876
**448**	NDRG4	Protein NDRG4	0.0078876
**449**	PAQR7	Membrane progestin receptor alpha	0.0078876
**450**	CHAC1	Glutathione-specific gamma-glutamylcyclotransferase 1	0.0078876
**451**	CPXM2	Inactive carboxypeptidase-like protein X2	0.0078876
**452**	FAAH2	Fatty-acid amide hydrolase 2	0.0079609
**453**	MAML3	Mastermind-like protein 3	0.007996
**454**	ASGR2	Asialoglycoprotein receptor 2	0.007996
**455**	NKIRAS1	NF-kappa-B inhibitor-interacting Ras-like protein 1	0.0080305
**456**	LEFTY2	Left-right determination factor 2	0.0080649
**457**	CORO7	Coronin-7	0.008094
**458**	KLHL29	Kelch-like protein 29	0.0081189
**459**	TMPRSS6	Transmembrane protease serine 6	0.0081971
**460**	CRISPLD1	Cysteine rich secretory protein LCCL domain containing 1	0.0082215
**461**	CIB4	Calcium and integrin binding family member 4.	0.0082964
**462**	H2AFB1	Histone H2A-Bbd type 1	0.0082979
**463**	TRIM24	Transcription intermediary factor 1-alpha	0.0083573
**464**	AMOTL1	Angiomotin-like protein 1	0.0083853
**465**	PLIN2	Perilipin-2	0.0083853
**466**	C3	Complement C3c alpha' chain fragment 1	0.0083946
**467**	PPP1R17	Protein phosphatase 1 regulatory subunit 17	0.0083961
**468**	RASAL1	RasGAP-activating-like protein 1	0.0084578
**469**	FOXK1	Forkhead box protein K1	0.0085051
**470**	COG2	Conserved oligomeric Golgi complex subunit 2	0.008565
**471**	NDFIP1	NEDD4 family-interacting protein 1	0.0086092
**472**	ANXA8L1	Annexin A8 like 1	0.0086248
**473**	RC3H2	Roquin-2	0.0086628
**474**	HIST1H2AL	Histone H2A type 1	0.0086755
**475**	TPST1	Protein-tyrosine sulfotransferase 1	0.0087119
**476**	ASB1	Ankyrin repeat and SOCS box protein 1	0.008736
**477**	TAS2R5	Taste receptor type 2 member 5	0.0087899
**478**	ARSG	Arylsulfatase G	0.0089389
**479**	SH3PXD2B	SH3 and PX domain-containing protein 2B	0.0089775
**480**	MSLN	Megakaryocyte-potentiating factor	0.0089775
**481**	AVIL	Advillin	0.0089775
**482**	SLAMF1	Signaling lymphocytic activation molecule	0.0089775
**483**	PHACTR4	Phosphatase and actin regulator 4	0.0089775
**484**	ABCD2	ATP-binding cassette sub-family D member 2	0.0089775
**485**	ERVW-1	Transmembrane protein	0.0089775
**486**	CHP1	Calcineurin B homologous protein 1	0.0089775

Table S4

487	PABPN1	Polyadenylate-binding protein 2	0.0089775
488	LHX8	LIM/homeobox protein Lhx8	0.0089775
489	NUP54	Nucleoporin p54	0.0089775
490	C22orf42	Uncharacterized protein C22orf42	0.0089775
491	CAPSL	Calcyphosin-like protein	0.0089775
492	CSH2	Chorionic somatomammotropin hormone 2	0.008984
493	MRPL2	Mitochondrial ribosomal protein L2	0.0090054
494	JKAMP	JNK1/MAPK8-associated membrane protein	0.0090439
495	FMNL3	Formin-like protein 3	0.009056
496	INPP5J	Phosphatidylinositol 4	0.0090731
497	CROCC	Rootletin	0.0091129
498	TMEM253	Transmembrane protein 253.	0.0091819
499	KIAA0922	Transmembrane protein 131-like	0.0092234
500	XPR1	Xenotropic and polytropic retrovirus receptor 1	0.0092237
501	NAA16	N-alpha-acetyltransferase 16	0.0092237
502	PLP2	Proteolipid protein 2	0.0092237
503	FANCA	Fanconi anemia group A protein	0.0092777
504	UHMK1	Serine/threonine-protein kinase Kist	0.009288
505	CDC14A	Dual specificity protein phosphatase CDC14A	0.0093137
506	PCBD2	Pterin-4-alpha-carbinolamine dehydratase 2	0.0093354
507	RGS20	Regulator of G-protein signaling 20	0.009373
508	ISLR	Immunoglobulin superfamily containing leucine rich repeat.	0.0093775
509	GRAP2	GRB2-related adapter protein 2	0.0093776
510	C5orf20	Dendritic cell nuclear protein 1	0.0094531
511	PUS10	Putative tRNA pseudouridine synthase Pus10	0.0094827
512	C12orf54	Uncharacterized protein C12orf54	0.0094987
513	DRG1	Developmentally-regulated GTP-binding protein 1	0.0094988
514	FBXL4	F-box and leucine rich repeat protein 4.	0.0095225
515	IQUB	IQ and ubiquitin-like domain-containing protein	0.0095326
516	PAWR	PRKC apoptosis WT1 regulator protein	0.0095378
517	FUNDC2	FUN14 domain containing 2	0.0095675
518	OLFM3	Noelin-3	0.0096516
519	PPM1L	Protein phosphatase 1L	0.0096567
520	DCTPP1	dCTP pyrophosphatase 1	0.009736
521	CUTC	Copper homeostasis protein cutC homolog	0.0097372
522	ADAMTSL5	ADAMTS-like protein 5	0.0097406
523	PSG4	Pregnancy specific beta-1-glycoprotein 4.	0.0097664
524	FIG4	Polyphosphoinositide phosphatase	0.0097956
525	CASP2	Caspase-2 subunit p12	0.0098131
526	DAO	D-amino-acid oxidase	0.0098138
527	APOL6	Apolipoprotein L6	0.009867
528	GPR182	G-protein coupled receptor 182	0.0098814
